# The Bidirectional Relationship Between Myocardial Infarction and Depression: Risk Factors, Mechanisms, and Interventions

**DOI:** 10.3390/biomedicines13112838

**Published:** 2025-11-20

**Authors:** Zhuorui Cui, Qiaoning Yang, Furong Yang, Yankai Yang, Xuexin Yang, Yanqiao Yu, Yajie Cai, Xiaodi Fan, Ruina Bai

**Affiliations:** 1 Graduate School, Beijing University of Chinese Medicine, Beijing 100029, China; 2 Xiyuan Hospital, China Academy of Chinese Medical Sciences, Beijing 100091, China; 3 National Clinical Research Center for Chinese Medicine Cardiology, Xiyuan Hospital, China Academy of Chinese Medical Sciences, Beijing 100091, China; 4 NMPA Key Laboratory for Clinical Research and Evaluation of Traditional Chinese Medicine, Beijing 100091, China; 5 Institute of Basic Medical Sciences, Xiyuan Hospital of China Academy of Chinese Medical Sciences, Beijing 100091, China; 6 Key Laboratory of Pharmacology of Chinese Materia Medica, Beijing 100091, China

**Keywords:** myocardial infarction, depression, bidirectional relationship, heart–brain axis, risk factor, mechanism, intervention

## Abstract

Myocardial infarction (MI) and depression exhibit a bidirectional relationship, in which patients with MI are more susceptible to depression, and individuals with depression face a heightened risk of MI. The two diseases are intricately intertwined via the heart–brain axis. Sex, age, lifestyle, social background, comorbidities, and genetics contribute to and affect the prognosis of this combined condition. Mechanisms involving the autonomic nervous system (ANS), hypothalamic–pituitary–adrenal (HPA) axis, inflammation, thrombosis, tryptophan metabolism, renin–angiotensin–aldosterone system (RAAS), endothelial dysfunction, microRNAs, and gut microbiota, as components of the heart–brain axis, have been implicated in the pathological link between MI and depression. This review outlines the common risk factors and potential mechanisms underlying this bidirectional relationship. It treats the comorbidities of MI and depression as a unified condition, relying on evidence from clinical trials and experimental studies that directly address both diseases together rather than extrapolating from separate studies on MI or depression alone. It also discusses current therapeutic approaches, including non-pharmacological interventions like psychotherapy and exercise, and pharmacological treatments with chemical or natural compounds. Finally, this review identifies significant gaps in the pathophysiology and clinical management of MI with depression, which warrant further investigation.

## 1. Introduction

Myocardial infarction (MI), which is characterised by myocardial necrosis due to acute sustained coronary ischaemia and hypoxia, remains the leading cause of death and disability worldwide [1]. Depression, which afflicts approximately 5.7% of adults worldwide, has emerged as one of the fastest-growing global disease burdens and is the single largest contributor to disability within mental disorders [1,2,3]. Mendelian randomization analysis demonstrated a significant bidirectional causal relationship between the two diseases [4].

About one-third of MI patients exhibit depression [4,5,6,7,8], a rate five times higher than that of the general population. Among them, 5–20% experience major depressive disorder (MDD) [9,10,11], while nearly 20% present with mild depressive symptoms [6]. Comorbid depression increases mortality risk by 41% in MI patients [3]. This elevated risk may be mediated by depression-related unhealthy behaviours, such as smoking, obesity, physical inactivity, and poor diet, thereby establishing a vicious cycle [12]. Depression is an independent risk factor for MI and significantly worsens prognosis [13]. Additionally, changes in depressive symptoms have been recognised as a predictor of incident cardiovascular diseases (CVD) [14]. Therefore, the comprehensive management of patients with comorbid MI and depression is critical for reducing the risk of future events.

**Figure 1:**
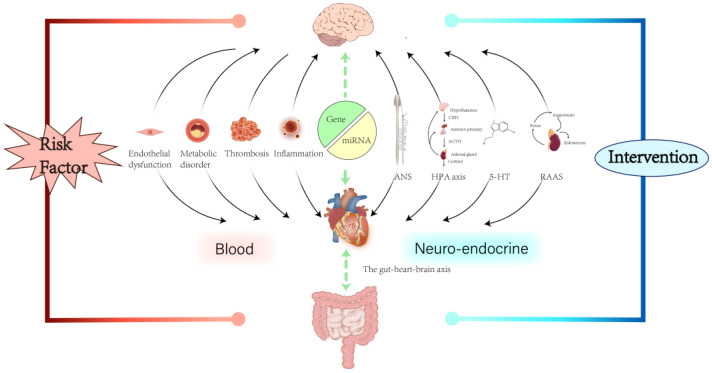
Both genes and miRNAs regulate gene expression and lie upstream in the pathogenesis of MI combined with depression, reflecting innate mechanisms. Communication pathways between the heart and brain occur primarily via the circulatory system and neuroendocrine signalling. Inflammation, thrombosis, metabolic disorders, and endothelial dysfunction are primarily blood-related, while the ANS, HPA axis, tryptophan metabolism, and RAAS are mainly neuroendocrine-related. The gut–heart–brain axis represents a distinct mechanism, separate from congenital, haematological, or neuroendocrine pathways. Except for endothelial dysfunction and thrombosis, which primarily reflect depression’s effect on MI, other mechanisms contribute to the bidirectional relationship between MI and depression. MiRNA, microRNA; MI, myocardial infarction; ANS, autonomic nervous system; HPA, hypothalamic–pituitary–adrenal; RAAS, renin–angiotensin–aldosterone system.

In recent years, increasing attention has been paid to the significant impact of MI combined with depression, prompting extensive research on potential risk factors, underlying pathophysiological mechanisms, and targeted interventions. Investigating the bidirectional relationship between MI and depression may shed light on the mechanisms driving this comorbidity as well as the development of the detrimental cycle between the two conditions. However, current research has largely focused on single-disease models, speculating on the interplay between MI and depression based on isolated mechanisms without sufficient comprehensive clinical and experimental evidence. In contrast, the present review considers MI and depression as a syndrome, detailing the associated risk factors and the intricate mechanisms underlying their interactions, spanning from the clinical level to the molecular scale. It further assesses interventions targeting comorbidities (Figure 1). This work not only summarises the well-established effect of depression on MI but also explores the effect of MI on depression, thereby complementing the bidirectional relationship. It aims to offer fresh perspectives to researchers in both cardiovascular and psychological fields, stimulate further inquiry, and improve prevention and treatment approaches for MI combined with depression.

## 2. Risk Factors

### 2.1. Sex and Age

Traditionally, male sex and older age have been recognised as risk factors for CVD. A global prospective study suggested a stronger link between depression and CVD risk in men [15], potentially due to lower treatment adherence [16]. However, female sex is a strong predictor of post-MI depression [17,18], with the prevalence of depression among women aged 18–44 years twice that in men in the same age group [19]. Women of all ages with depression or those experiencing higher stress have nearly double the incidence of MI compared with men [20,21], potentially influenced by their non-adherence to secondary prevention measures [22]. Similarly, depression alters the relationship between age and CVD. When combined with depression, the occurrence of abnormal corrected QT intervals (a marker of cardiac autonomic dysfunction) is 10.6 times higher in the younger population (<65 years) than in the older population (>65 years) [23]. Younger patients (<30 years) with depression face a higher hazard of major adverse cardiovascular events (MACE) [24]. The risk of post-MI depression is tripled in younger patients (<55 years) but is reduced by 54% in older patients (>70 years) [25]. However, A Scottish study reported that elderly patients with MI and MDD experience higher in-hospital mortality, potentially because of worse physical health and greater comorbidities [26].

### 2.2. Lifestyle

The primary lifestyle factors influencing comorbidities include poor nutrition, alcohol consumption, smoking, and sleep disturbances. Depression increases the risk of MACE by 82% in individuals with poor diets, particularly diets low in vegetables and polyunsaturated fatty acids [27]. Poor diet can also lead to malnutrition. Comorbid malnutrition in coronary artery disease (CAD) combined with depression patients raises the risk of CVD-related death and adverse outcomes by 80% [28]. Similarly, reduced fruit and vegetable intake increases depression prevalence by 1.68-fold in patients with coronary heart disease (CHD) [29]. Patients with depression who drink alcohol have a 1.4-fold higher risk of MI than nondrinkers [30], and those with a history of alcohol abuse are more likely to be hospitalised for depression following MI [25]. Smoking mediates 24.9% of the effects of depression on MI [31], substantially increasing the risk of MI in patients with depression. However, adjusting for smoking reduced this relationship [32]. Smoking is also associated with higher rates of depression and rehospitalisation in patients with CAD [25,33,34], doubling the risk of depression during recovery after an acute cardiac event [35]. By contrast, smoking cessation improves or even resolves depressive symptoms in patients with acute coronary syndrome (ACS) comorbid with depression [36,37]. Sleep disorders and depression synergistically increase the risk of CHD morbidity and mortality [38,39], while normal sleep patterns reduce antidepressant use by 24% in patients with CVD [40]. Low physical activity and irregular exercise increase the risk of depression in patients with CAD [17,41], but have a minimal impact on the risk of ischaemic heart disease (IHD) in individuals with depression [42].

### 2.3. Social Background

Globally, CVD risk among patients with depression is significantly higher in urban areas than in rural settings [43]. Elevated crime rates in a given Finnish area strengthen the association between depression and CHD [44]. Notably, economic status appears to have little impact on CVD risk after depression [43]. Educational level and work-related stress independently contribute to cardiac risk irrespective of depressive status [45,46]. However, unemployment and lower educational levels markedly increase the likelihood of depression among German patients who have experienced an acute cardiac event [47]. Higher educational attainment reduces depression prevalence in patients with ACS in Trinidad and Tobago by 28% [34]. In Australia, Financial stress increases the risk of depression during MI recovery by 4–5-fold compared with individuals with less economic strain. Lower socioeconomic status nearly doubles the risk of depression compared with individuals with higher status [35]. High stress, except financial stress, more than doubles the risk of moderate-to-severe depression in patients with CHD. Among various stressors, work-related factors, such as job strain, effort-reward imbalance, job insecurity, long working hours, and bullying, significantly contribute to the burden of depression and CHD, with attributable fractions of 17–35% for depression and 5–11% for CHD [29,48]. Importantly, variations in social background, such as worldviews and income levels, lead to divergent perceptions of depression. Consequently, caution is warranted when considering the generalizability of these research findings.

### 2.4. Complications

Diabetes, metabolic syndrome, and anxiety are strongly associated with an increased risk of cardiovascular death in patients with depression, and a high incidence of depression in individuals with MI [49,50]. Genetic liability to depression is associated with higher MI risk, with 24.1% mediated by diabetes [31], and the combination of diabetes and depression amplifies cardiovascular mortality risk [51,52], with diabetes increasing the CVD risk by 6.5-fold in patients with depression [53]. Diabetes also increases the incidence of depression by 1.3-fold in patients with MI, particularly during the later recovery stages [35,54]. Elevated haemoglobin A1c levels, a key diabetes marker, worsen the prognosis of patients with comorbid CHD depression [55]. The correlation between depression and cardiovascular events is strengthened by several metabolic risk factors [56], with central obesity and lipid imbalances playing significant mediating roles [32,57,58]. Poor adherence to lipid-lowering therapy [59], low-density lipoprotein (LDL) L5 and pro-protein convertase subtilisin/kexin type 9 (PCSK9)-related lipid dysregulation, and insulin resistance may underlie this relationship [60,61]. Overweight/obesity not only increases the risk of IHD by 1.6-fold in patients with depression [42], but also increases the likelihood of depression during early MI recovery [35]. Hyperlipidaemia increases the risk of depression by 1.5-fold in Chinese patients with CHD [33]. Conversely, statin therapy can reduce the risk of depression [62]. Abnormal triglyceride metabolism may mediate the interaction between MI and depression [63]. Anxiety commonly coexists with depression and causes a further reduction in high-frequency heart rate variability (HRV) and additional impairment of parasympathetic function in depressed patients [64,65]. Women are particularly vulnerable to comorbid anxiety-depression, which significantly increases their risks of developing chronic diseases [66].

### 2.5. Genetics

Genetic predisposition is a significant risk factor for comorbid depression and MI. The *FK506 binding protein 51 (FKBP5) C allele*, previously associated with psychiatric disorders such as depression, leads to increased depressive symptoms in patients with CHD who have a history of MI or CAD [67]. However, this allele does not appear to increase the risk of depression in German patients newly diagnosed with CHD [68]. The *apelin receptor (APLNR) rs9943582* polymorphism has been implicated in increased CAD risk [69,70], and its *C allele* has been linked to both depression prevalence and severity in patients with CHD [71]. When higher genetic susceptibility to CHD is combined with severe depression, the risk of developing CHD increases 2.7-fold [72].

## 3. Mechanisms

### 3.1. Autonomic Nervous System

Autonomic nervous system (ANS) dysfunction is characterised by hyperactivation of the sympathetic nervous system (SNS) and reduction in parasympathetic nervous system (PNS) signalling. The SNS primarily increases heart rate (HR) and myocardial contractility through catecholamine release (mainly epinephrine and norepinephrine (NE)), whereas the PNS counteracts this effect via acetylcholine (ACh) release. Thus, the ANS is one of the key systems of heart–brain connections. HRV is the main indicator of ANS dysfunction and can be driven by both MI and depression [73]. Proteins such as sigma-1 receptor (S1R) and G protein-coupled receptor kinase-2 (GRK2) may play a regulatory role in modulating ANS.

#### 3.1.1. Effect of ANS Dysfunction After MI on Depression

ANS dysfunction or dysplasia can lead to the development of psychiatric disorders [74,75,76,77,78], and MI can induce ANS dysfunction, contributing to depression onset. Patients post-MI with depression exhibit significantly lower HRV than those without depression [79]. Interventions such as HRV biofeedback can enhance HRV, modulate ANS function, reduce depressive symptoms, and lower the risk of readmission in MI patients [80,81,82].

GRK2 may represent a key protein linking ANS dysfunction, MI, and depression. Experimental evidence from post-MI heart failure models demonstrates that interventions increasing vagal efferent activity (e.g., optogenetic stimulation) reduce myocardial GRK2 expression. Inhibition of GRK2 improves cardiomyocyte function; modulates β-adrenoceptor signalling; restores cardiac volume, left ventricular ejection fractions (LVEF), and systemic haemodynamics; reduces HR and blood pressure (BP); and improves exercise capacity [83,84,85]. In patients with MI comorbid with depression, GRK2 expression in peripheral lymphocytes is inversely correlated with HRV and positively correlated with depression scores. Treatment with the GRK2 inhibitor paroxetine significantly improves depression scores, HRV, and LVEF, with greater improvements in cardiac function compared with fluoxetine [86]. These findings suggest that GRK2 overexpression may play a key role in ANS dysfunction, leading to impaired cardiac function and the exacerbation of depression. However, whether the effects of paroxetine are solely due to GRK2 inhibition, or whether it acts as a selective serotonin reuptake inhibitor (SSRI) remains unclear. Therefore, further research is required to elucidate the mechanisms by which GRK2 contributes to ANS dysfunction and MI-associated depression.

#### 3.1.2. Effect of ANS Dysfunction After Depression on MI

Depression also contributes to ANS dysfunction, thereby affecting cardiovascular activity. Patients with MDD and rats exposed to chronic social defeat stress exhibit reduced HRV and elevated HR and BP [87,88,89]. The elevation of circulating levels of adrenaline and noradrenaline in depressed animals may lead to increased cardiac fibrosis, reduced cardiomyocyte counts, and atrial electrical instability. The recovery of left ventricular end-diastolic pressure (LVEDP) after ischemia/reperfusion (I/R) injury is significantly delayed [90], and pulsed electrical stimulation is more likely to induce atrial fibrillation [91]. This phenomenon may be associated with decreased S1R expression in the heart and hippocampus of depression animals. Administration of the selective S1 receptor agonist SA4503 can improve cardiac function [91]. These findings suggest that the downregulation of S1R in depression may act as an upstream protein involved in ANS dysfunction, contributing to cardiac damage.

Although the results of numerous studies suggest that the ANS is a key mediator in the interaction between MI and depression and that ANS dysfunction is a potential mechanism underlying this comorbidity, findings from the large Netherlands Study of Depression and Anxiety cohort challenge this view. After adjusting for antidepressant use, the association between cardiac ANS dysfunction and depression became less significant, implying that the previously reported link may have been confounded by antidepressant effects, rather than reflecting a true relationship [92]. Moreover, sex differences may play a critical role in ANS regulation post-MI. Female mice exhibit less vagal dysfunction and greater central vagal activation than male mice following MI. In male mice with MI, administration of 17β-estradiol significantly attenuates cardiac parasympathetic dysfunction; enhances vagal baroreflex sensitivity; and reduces vagal ganglionic glutamatergic remodelling, oxidative stress, and mitochondrial dysfunction [93]. These findings suggest that oestrogen modulates vagal remodelling after MI, which could partly explain the lower incidence of ventricular arrhythmias and sudden cardiac death post-MI in women compared with men. However, most current studies predominantly use male animal models, and sex differences are frequently inadequately accounted for in clinical studies. Therefore, further investigations are needed to clarify how sex influences ANS function in the context of MI combined with depression.

### 3.2. Hypothalamic–Pituitary–Adrenal Axis

The Hypothalamic–Pituitary–Adrenal (HPA), a crucial component of the neuroendocrine system, plays a central role in the interplay between MI and depression. This axis functions through hypothalamic corticotropin-releasing hormone (CRH), which prompts pituitary adrenocorticotropic hormone (ACTH) release, leading to glucocorticoid (GC) production in the adrenal cortex. There are two main types of cortisol receptors (CRs)—mineralocorticoid receptors (MRs) and glucocorticoid receptors (GRs). Normally, GC binds to high-affinity MRs; however, during stress or GC elevation, low-affinity GRs are activated to regulate GC via negative feedback. However, chronic GC elevation can reduce GR expression [94], impairing the negative feedback regulation of the HPA axis. Experimental studies have demonstrated a strong link between MI combined with depression and disruptions in the HPA axis feedback mechanism, as well as abnormal CR signalling [95]. In addition, the two diseases may mutually influence each other through dysregulation of the HPA axis [96].

#### 3.2.1. Effect of HPA Axis Dysfunction After MI on Depression

ACS triggers activation of the HPA axis, leading to abnormal GC elevation or a flattened diurnal cortisol slope (DCS), both of which are linked to mood disorders, including depression and suicidal ideation [97,98]. For example, a steeper DCS suggests a 34% reduction in depression risk 12 months after coronary artery bypass grafting [99]. Interestingly, elevated GC levels and flat DCS in patients with MI seem to be more related to the duration of depression than to its severity or prior history [98]. MI has been shown to be a key trigger of HPA axis dysregulation when the MR/GR balance is disrupted. Baseline serum GC levels do not change significantly in MR/GR-KO mice, but MI-induced HPA axis Dysregulation and depression behaviour are more severe than in normal groups [100]. Dysregulation of the HPA axis after MI is accompanied by abnormally elevated levels of markers of cardiac injury and glial fibrillary acidic protein (GFAP) in the hippocampus, reflecting astrocyte activation, neuronal damage and cardiac injury [101,102]. Administration of GC further upregulates these indicators, exacerbating depression, cognitive deficits and cardiac dysfunction [94,101]. Dysregulation of the HPA axis after MI and subsequent depression may be influenced by inflammatory factors, particularly Interleukin (IL)-12A and tumour necrosis factor (TNF)-α, but not IL-1β and IL-6 [102]. Elevated methylation levels of nuclear receptor subfamily 3 group C member 1 (NR3C1) modulate GR expression, significantly increasing the risk of depression within 2 weeks of ACS [103].

#### 3.2.2. Effects of HPA Axis Dysfunction After Depression on MI

Similarly, depression induces dysfunction of the HPA axis, leading to abnormal GC levels, which in turn elevate CVD risk [104]. Elevated GC or flat DCS profiles in depression patients are accompanied by cardiovascular system-related abnormalities such as elevated diastolic blood pressure, reduced high-density lipoprotein cholesterol (HDL-C), and increased blood glucose levels [105,106,107]. In depression mice, GC levels elevate, Toll-like receptor 4 (TLR4) and nuclear factor kappa B (NF-κB) expression in bone marrow mononuclear cells show upregulated, as well as intima-media thickness, HR, plasma cholesterol, triglycerides, and low-density lipoprotein (LDL)-to-HDL ratios are increased. GR knockdown of GR reversed the above phenotypes [108,109]. These findings suggest that the GR-mediated NF-κB/TLR4 signalling pathway may play a crucial role in the connection of depression, HPA axis and cardiovascular system.

The “corticosteroid receptor (CR) hypothesis of depression”, proposed by Florian Holsboer in 2000, posits that impaired CR signalling is a key mechanism in the pathogenesis of depression [110]. The above studies suggest that GR dysfunction not only contributes to depression onset following MI [100,103], but also mediates the effects of depression on the cardiovascular system [108]. Thus CR signalling might be a key pathological mechanism in MI combined with depression.

However, studies on CHD combined with depression have not consistently demonstrated hyperactivity of the HPA axis. Lower GC levels in patients with CHD have been linked to depression and mental fatigue [111,112]. Chronic emotional stress can lead to HPA axis dysfunction, decreased GC levels, increased cardiac load, and the risk of silent myocardial ischaemia, particularly in Black African men [113]. Comorbid conditions may also influence the GC-depression relationship in patients with MI, with a positive correlation found only when post-traumatic stress disorder is present [114]. Additionally, dysregulation of the HPA axis seems more relevant to post-ACS depression than to post-depressive ACS. Patients diagnosed with MDD after ACS show a sluggish cortisol awakening response (CAR), whereas a history of depression is not associated with CAR abnormalities [115]. A 2020 two-way Mendelian randomization study concluded that GC levels were not causally linked to IHD or CVD risk factors (e.g., obesity, glucose levels, BP, and lipids), and vice versa [116]. Therefore, the role of GC in the relationship between depression and MI remains unclear.

Overall, the dysregulation of the HPA axis may partially explain the interaction between MI and depression. MI triggers depression through hippocampal damage mediated by the HPA axis, which, in turn, increases the risk of MI. However, further research is required to clarify this relationship.

**Figure 2:**
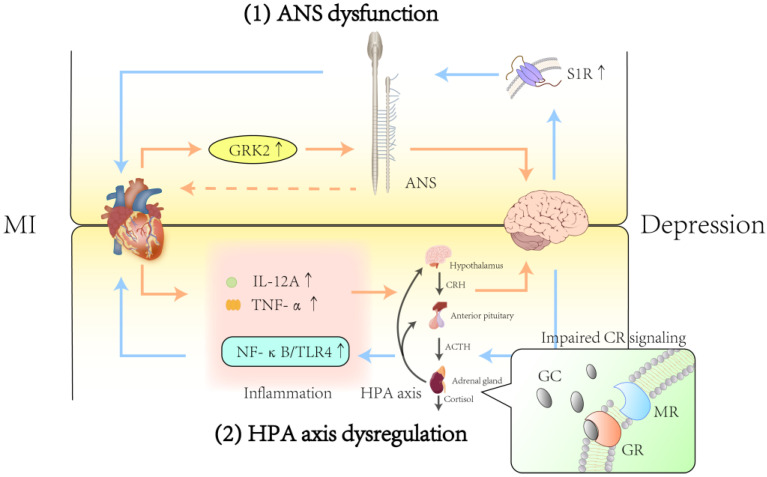
The ANS and HPA axis are critical mediators of the bidirectional relationship between MI and depression. (1) Post-infarction overexpression of GRK2 contributes to ANS dysfunction, impairing cardiac function and exacerbating depression. Depression-related downregulation of the S1R further impairs ANS function, damages cardiac structure, and accelerates CVD progression. (2) IL-12A and TNF-α, elevated following MI, may promote depression through impairing HPA axis. Depression activates the HPA axis, with the GR-mediated NF-κB/TLR4 signalling pathway playing a pivotal role in cardiovascular pathology. In addition, impaired CR signalling might be a key pathological mechanism in MI combined with depression. MI, myocardial infarction; GRK2: G protein-coupled receptor kinase 2; S1R: sigma-1 receptor; CVD: cardiovascular disease; IL-12A, interleukin-12A; TNF-α: tumour necrosis factor; TLR4, toll-like receptor 4; GC: glucocorticoid; GR: glucocorticoid receptor; MR: mineralocorticoid receptor; CR: corticosteroid receptor.

The mechanisms for the ANS and HPA axis are summarised in Figure 2.

### 3.3. Inflammation

**Figure 3:**
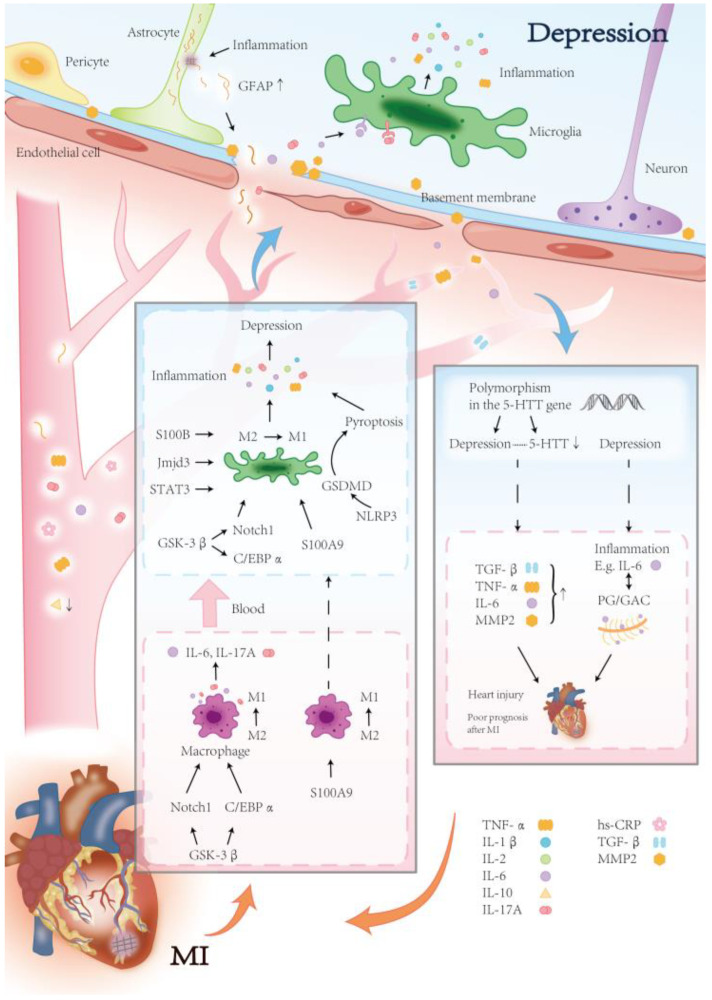
Inflammatory factors mediate heart–brain communication, a process exacerbated by BBB disruption. The intense inflammatory response following MI triggers neuroinflammation and contributes to depression onset. Conversely, inflammation linked to depression worsens MI and impairs prognosis. The impact of MI on depression is primarily driven by macrophage and microglial activation, potentially mediated by GSK-3β, NLRP3 inflammasomes, calgranulin B or migration inhibitory factor-related protein 14 S100A9, S100B, JMJD3 and STAT3. Regarding the effect of depression on infarction, the role of inflammation is regulated by tryptophan metabolism. Depression-induced inflammation is associated with PG and GAG interactions in the heart, leading to cardiac damage. BBB: blood–brain barrier; MI, myocardial infarction; GSK-3β, glycogen synthase kinase 3 beta; NLRP3, NOD-like receptor thermal protein domain associated protein 3; S100A9, s100 calcium binding protein A9; S100B, s100 calcium binding protein B; JMJD3, jumonji domain-containing protein 3; STAT3, signal transducer and activator of transcription 3; PG: proteoglycan; GAG: glycosaminoglycan; GFAP: glial fibrillary acidic protein.

Immunoinflammatory processes are increasingly recognised as important mechanisms in MI combined with depression. Following MI, a robust systemic inflammatory response is well established [117,118], and individuals with depression consistently exhibit elevated levels of inflammatory markers [119]. Inflammation may play a crucial role in mediating both the impact of MI on depression and the influence of depression on MI. This mechanism is shown in Figure 3.

#### 3.3.1. Effect of Inflammation After MI on Depression

After MI, apoptotic cardiomyocytes initiate a dramatic inflammatory cascade. Inflammation compromises the integrity of the blood–brain barrier (BBB) by upregulating matrix metalloproteinases (MMPs), damaging the vascular endothelium, and increasing cellular permeability [120,121]. Disruption of the BBB allows pro-inflammatory molecules to invade the central nervous system (CNS), activate microglia, and drive neuroinflammation, inducing or exacerbating depression, which may be manifested as a sustained elevation of glial fibrillary acidic protein (GFAP) levels [122].

Inflammation levels in CVD patients are strongly associated with depression. Elevated levels of high-sensitivity CRP (hs-CRP) in patients undergoing percutaneous coronary intervention (PCI), NOD-like receptor thermal protein domain associated protein 3 (NLRP3) in patients with ST-segment elevation myocardial infarction (STEMI), and TNF-α and IL-17A in CHD patients have all been associated with depression [123,124,125]. However, IL-6, IL-18, and IL-1β levels are not significantly correlated with depression in patients with CHD and STEMI [123,124]. A single inflammatory marker may be influenced by various factors, making a composite indicator a more reliable measure. For instance, in patients with ACS undergoing their first PCI, admission neutrophil to lymphocyte ratio (NLR) levels were positively correlated with depression severity 1 month post-procedure. This marker is clinically accessible and may be a key predictor of depression following PCI [126]. Experimental studies have demonstrated that the application of a pro-inflammatory factor synthesis inhibitor reduces the levels of inflammatory markers (e.g., IL-1β, IL-2, IL-6, and TNF-α) in serum and brain regions including the paraventricular nucleus and prefrontal cortex of rats with MI, and alleviates depressive-like behaviours [127].

Research on the inflammatory mechanisms underlying post-MI depression has largely focused on microglial activation. Microglia, the primary immune cells of the CNS, are quickly activated by pathological stimuli and exhibit two primary phenotypes: M1, which promotes inflammation and releases pro-inflammatory mediators, and M2, which facilitates tissue repair and regeneration. In post-MI depressed rats, treatment with the antibiotic minocycline inhibits systemic and brain inflammation, reduces microglial overactivation and infarct size, increases sucrose preference, reduces immobility in the forced swim test, and improves cardiac function by increasing ejection fraction and decreasing LVEDP. These results highlight the role of microglial activation and inflammation in depression and cardiac dysfunction following MI [128]. Microglial activation is regulated by numerous proteins, including glycogen synthase kinase 3 beta (GSK-3β), NLRP3 inflammasomes, s100 calcium binding protein A9 (S100A9), s100 calcium binding protein B (S100B), jumonji domain-containing protein 3 (JMJD3), and signal transducer and activator of transcription 3 (STAT3). The results of animal and human studies have shown that the numbers of M1-type macrophages increase while those of M2-type macrophages decrease after MI. IL-6 and IL-17A produced by M1-type macrophages cross the BBB and activate M1-type microglia in the brain, which in turn release pro-inflammatory factors to induce depression. Macrophage/microglia polarisation may be mediated through the GSK-3β/notch receptor 1 (Notch1) and GSK-3β/CCAAT/enhancer-binding protein alpha (C/EBPα) signalling pathways. LiCl, a GSK-3β inhibitor, reverses this phenomenon [129]. NLRP3-mediated gasdermin-D (GSDMD)-induced microglial pyroptosis has also been identified as a key pathway in MI and depression [130]. Furthermore, in the heart and hippocampus of MI rats, S100A9 expression is elevated; the number of CD68+ macrophages and Iba1+ microglia is increased; and the levels of pro-inflammatory factors like IL-1β and TNF-α are heightened. Treatment with an S100A9 inhibitor reverses these changes, ameliorates cardiac and neurological pathology, and alleviates depression-like behaviour [131,132]. Thus, S100A9-mediated macrophage/microglial inflammation is pivotal in post-MI depression. Similarly, elevated S100B, JMJD3, and STAT3 expression in the brain tissues of mice with MI or MI combined with heart failure induces microglial activation and increases pro-inflammatory cytokines. Inhibition of these proteins effectively reverses pathological changes and alleviates depressive symptoms [133,134,135,136,137].

#### 3.3.2. Effects of Inflammation After Depression on MI

Depression-induced inflammation can also trigger cardiovascular events, but the mechanism is poorly understood. The Systemic Immune Inflammation Index (SII), which integrates neutrophil, lymphocyte, and platelet counts, is a useful marker for assessing immune inflammation. A large cohort study from the UK Biobank involving 176,428 adults without CHD found that depression increased the risk of premature CHD by 72%, with SII mediating 2.7% of the association between the two diseases [58]. In individuals with prior MI, depression increases the likelihood of recurrent myocardial infarction (MIR), which is positively correlated with elevated hs-CRP levels [138]. Sustained inflammation after emotional distress such as anxiety and depression also heightens the risk of MI and worsens prognosis [139]. In patients with STEMI, emotional stress upregulates macrophage migration inhibitory factor (MIF), exacerbating inflammation, thus promoting atherosclerotic plaque rupture and MI progression [140]. MIF- adenosine 5′-monophosphate-activated protein kinase (AMPK) signalling may be involved [141]. Moreover, preoperative depressive and anxiety symptoms in patients undergoing cardiac surgery are associated with elevated postoperative CRP levels [142], which can impede recovery [143].

Depression is influenced by polymorphisms in the 5-HT transporter (5-HTT or SERT) genes. Individuals with the short allele are more prone to depression under stress and their 5-HTT expression is generally lower [144,145,146]. MI 5-HTT knockout (*5-HTT-/-*) mice show behavioural deficits and significantly elevated transforming growth factor-β (TGF-β), TNF-α, IL-6, and MMP-2 levels, impairing the early healing in MI. All *5-HTT-/-* mice succumbed when the infarct size exceeded 30% and heart failure occurred, whereas *5-HTT+/-* and *5-HTT+/+* mice survived despite having more severe infarcts, likely due to differences in inflammatory response [147]. Proteoglycans (PGs) are non-fibrous components of the heart that consist of core proteins with attached glycosaminoglycan (GAG) chains. PG/GAG structural and functional changes are associated with heart disease. Depressed mice show altered GAG profiles, with increases of 17.9% and 35.3% in heparan sulphate (HS) and chondroitin sulphate (CS), respectively [148]. HS and CS can bind to pro-inflammatory factors, modulate inflammatory responses, and increase IL-6 mRNA expression [148,149,150]. Meanwhile, left ventricular wall thickness and cardiomyocyte cross-sectional area are also increased in these mice [148]. Thus, depression may negatively affect the heart through interactions between inflammation and cardiac PGs/GAGs.

Some studies have suggested the association between post-MI depression and immunosuppression. For instance, myeloperoxidase (MPO), a marker of innate immune activity, is significantly lower in post-MI patients with depression at baseline and 6 months. A low baseline MPO level was a significant predictor of depression development after MI at 6 months [151], in contrast to the excessive immune inflammation that is commonly observed. Additionally, depression may not directly influence cardiac inflammation, as no changes are detected in cardiac biomarkers such as high-sensitivity cardiac troponin I, N-terminal pro-B-type natriuretic peptide, Ischemia-modified albumin, or cardiac IL-1β in depressed rats [152]. However, it cannot be excluded that the results may be influenced by the degree of depression. Conversely, depression may worsen long-term outcomes and sudden cardiovascular events in patients with CVD through complex mechanisms. Many studies investigating the inflammation-mediated mechanisms of post-MI depression have focused on individual proteins. Transcriptomic analyses have identified different pivotal differentially expressed genes shared between MI and depression (e.g., cluster of differentiation 24, cystatin A, exostosin like glycosyltransferase 3, ribosomal protein S7, solute carrier family 25 member 5, zinc finger matrin-type 3, Toll-like receptor 2, HP, intercellular adhesion molecule 1, lipocalin-2, lactotransferrin, versican, S100A9, and NK-κB inhibitor alpha), all of which are associated with inflammation [153,154]. However, most candidate proteins have not been validated. Thus, while the role of inflammation in MI combined with depression is widely accepted, further in-depth studies are required to elucidate the upstream and downstream pathways of the relevant proteins, and to determine the differential roles of inflammation at different stages of the disease.

### 3.4. Platelet Activation and Coagulation Activation

The excessive activation of platelets and the coagulation system can lead to thrombosis, increasing the risk of CVD. Therefore, antiplatelet and anticoagulant therapies are routinely employed for secondary prevention in patients with MI, provided there are no contraindications. Emerging evidence suggests that depression may catalyse platelet activation and coagulation.

Anxiety-depressive syndrome significantly increases the average platelet count, platelet distribution width, and mean platelet volume in patients with CHD, reflecting enhanced platelet aggregation activity [155]. Depression severity also influences coagulation-related factors, including tissue factor (TF) and D-dimer concentrations, in patients with MI [156,157]. This hypercoagulable state caused by depression may be regulated by 5-hydroxytryptamine (5-HT) and the HPA axis, which in turn increases the risk of CVD due to the close association between thrombosis and CVD. In individuals with depression, reduced 5-HT levels lead to the compensatory upregulation of 5-HT receptors on the platelet surface, sensitising platelets to activation [158]. The administration of SSRIs helps restore 5-HT levels, thereby exerting antiplatelet and profibrinolytic effects [159]. For instance, pretreatment with varying concentrations of sertraline (an SSRI) and its primary hepatic metabolite, N-nitrosodimethylamine, results in dose-dependent inhibition of platelet aggregation and decreases the surface expression of thrombosis-related proteins [160]. D-dimer levels were 73 ng/mL higher in patients with MI with a Beck Depression Inventory score of ≥6 compared with patients with a score of <6. However, this positive association was significant only when patient cortisol levels were high [156].

The *brain-derived neurotrophic factor (BDNF) Val66Met* gene polymorphism is an innate factor that influences the interplay between depression, thrombosis, and CVD risk. The Met allele (*BDNF ^Met^*) may reduce BDNF levels and increase the risk of depression [161]. For example, *BDNF ^Met/Met^* mice display a depression-like phenotype [162]. The number of Met alleles is also positively correlated with the incidence of MACE within 6 months post-MI [163]. Individuals carrying *BDNF ^Met^* have an 85.7% higher risk of MI compared with those carrying *BDNF ^Val/Val^* [162]. Cardiac magnetic resonance imaging, collagen staining, and immunofluorescence revealed severe cardiac remodelling in *BDNF ^Met/Met^* mice post-MI [164]. Additionally, *BDNF ^Met/Met^* increases the risk of thrombotic events in patients with CVD and depression [162,165]. Among patients with CAD, the percentage of proplatelets released from megakaryocytes is nearly twice as high in those with *BDNF ^Met/Met^* compared with *BDNF ^Val/Val^* [165]. *BDNF ^Met/Met^* depressed mice are more prone to FeCl_3_-induced carotid artery thrombosis and exhibit higher mortality following collagen/adrenergic-induced pulmonary embolism. Proteomic analyses of aortic supernatants from these mice show reduced sirtuin 1 (SIRT1) levels, increased sortilin related VPS10 domain containing receptor 2 (SorCS2) expression and activity, elevated TF and gelsolin levels (both involved in coagulation), and increased pro-inflammatory α1-antitrypsin (A1AT) levels. In vivo experiments using the deacetylase inhibitor sirtinol to inhibit SIRT1 in wild-type mice, and in vitro experiments using SIRT1 activators and SorCS2 siRNA, have both confirmed that *BDNF ^Met^* regulates coagulation and inflammation, promotes thrombosis, and increases CVD risk through the SIRT1/SorCS2 pathway [162].

Another experimental study demonstrated a link between *BDNF ^Met^* and the NE/α2A-adrenergic receptor (α2A-ADR) pathway. In novelty-suppressed feeding and FeCl_3_-induced arterial thrombosis models, *BDNF ^Met/Met^* mice display significant behavioural deficits, reduced hippocampal BDNF levels, and rapid carotid artery thrombosis, accompanied by elevated NE levels in the bone marrow and plasma. Treatment with desipramine, a NE reuptake inhibitor, reversed these effects. Further in vitro aortic assays and HeLa cell culture confirmed *BDNF ^Met^* enhanced procoagulant activity and increased the sensitivity to the procoagulant effect of NE. Similarly, α2A-ADR was increased in *BDNF ^Met^* mice, cells, and patients with CAD. Stimulation of megakaryocytes from healthy subjects with *pro-BDNF ^Met^* and NE significantly upregulated α2A-ADR expression and enhanced platelet release. Administration of the α2-ADR antagonist Rauwolscine reversed this effect and prevented arterial thrombosis [165]. These findings suggest that the NE/α2A-ADR represents a key pathway mediating the impact of *BDNF ^Met^* on depression, thrombosis, and CVD risk.

Although most studies suggest that the prevalence of depression is higher among *BDNF ^Met^* carriers, no study has specifically examined the frequency of this allele in depressed populations. Given the high rate of *BDNF ^Met^* carriage in individuals with depression and the established link between *BDNF ^Met^*, thrombosis, and MI, it is highly likely to be a risk gene for post-depression MI. This raises the question regarding the necessity of classifying patients with depression into *BDNF ^Met^* and non- *BDNF ^Met^* subtypes, as the underlying mechanisms influencing CVD, such as MI, could differ between them. However, some evidence suggests that MDD in White populations or males is more primarily influenced by *BDNF Val66Met* polymorphism [166,167]. Therefore, further in-depth studies are required to explore the role of the *BDNF Val66Met* polymorphism and its impact on thrombosis in patients with MI and depression.

However, some studies have not observed the correlation between depression scores and plasma marker levels related to coagulation and fibrinolysis 3 months after MI onset [168]. In addition, fibrinogen does not appear to mediate the relationship between depression and CHD risk [169,170], and the impact of platelet overactivation on left ventricular function in patients with ACS does not seem to be linked to depression [171]. These findings suggest that thrombosis may not be the primary mechanism by which depression influences MI outcomes. Interestingly, impaired platelet cytoplasmic and mitochondrial functions in patients with ACS with comorbid depression or anxiety have been reported. After 12 months of follow-up, the incidence of cardiovascular complications in these patients was double that in those without comorbidities [172]. This finding indicates that depression and anxiety may impair rather than enhance platelet activity, contributing to worse cardiovascular outcomes.

Current studies provide limited evidence suggesting that MI increases the risk of depression through coagulation or platelet activation. Depression is likely to contribute to an elevated risk of thrombosis, thereby influencing CVD prognosis. However, more conclusive evidence is required to establish this causal relationship. The inconsistent findings across studies regarding the impact of various platelet and coagulation markers on patients with comorbid MI and depression may be influenced by factors such as patient sex, age, and ethnicity, as well as study design. Larger and more comprehensive studies are required to clarify these associations.

### 3.5. Tryptophan Metabolism

5-HT, a key neurotransmitter derived from tryptophan, is widely distributed in the brain, gut, and platelets, and serves as a crucial mediator between the CNS and the peripheral system. Its reuptake and homeostasis regulation are controlled by 5-HTT.

Abnormal tryptophan metabolism has been observed in the serum and brain tissues of patients and animal models of MI with comorbid depression. 5-HT levels are reduced in the serum and brain tissue [131,173,174,175]. 5-HT2A receptors are decreased in brain tissue [175,176]. Abnormal plasma-free L-tryptophan (L-Trp) scores and intensity-dependent auditory evoked potentials reflect reduced serotonergic neurotransmission [177]. Additionally, the mRNA expression of tryptophan metabolism-related enzymes, such as indoleamine 2,3-dioxygenase 1, kynurenine 3-monooxygenase, and kynureninase (KYNU), is increased in the hippocampus [173]; kynurenine (KYN) levels are elevated in the serum; and the KYN-to-tryptophan ratio is increased [134]. These metabolic disturbances are associated with both depression and CVD risk. The reduction in 5-HT or changes in its receptor function may promote the development of depression, with evidence extending back approximately five decades [178]. Transcriptomic analyses have suggested the critical role of tryptophan metabolism in atherosclerosis-induced depression, with genes such as caspase-1 and MMP-9, identified as key players [57]. In addition to its role in mood regulation, 5-HT significantly influences atherosclerosis, MI, heart failure, and hypertension [158,179,180,181,182]. Abnormalities in 5-HT and related proteins in the serum and platelets of individuals with depression or animal models may have detrimental effects on the cardiovascular system [183,184].

The link between tryptophan metabolism, MI, and depression may involve dysregulation of the HPA axis, inflammation, and thrombosis. In rodent models of depression combined with MI or chronic heart failure post-MI, disturbances in tryptophan metabolism are accompanied by abnormalities in the HPA axis, including increased hypothalamic CRH expression [134], decreased serum ACTH and GC levels [173], and reduced hippocampal GR expression [134]. Inflammation, potentially mediated by S100A9, GSK-3β, TNF-α/TNF-α Receptor (TNFR)/NF-κB signalling pathway or neutrophil degranulation, has been observed in the serum, hippocampus, and heart of these animals. The inflammatory response may interact with 5-HT, increasing the risk of depression or amplifying inflammation in infarcted cardiac tissue [129,131,134,173,185,186]. In addition, individuals carrying the *short allele of 5-HTT* express less 5-HTT and are more susceptible to depression [187]. The resulting reduction in 5-HTT expression may further enhance the autoimmune inflammatory response and worsen cardiac outcomes [144,145,147]. Moreover, reduced 5-HT may increase the risk of CVD by promoting platelet aggregation, vasoconstriction in diseased coronary arteries, and thrombosis [158,159,160,188]. SSRIs, which improve depression, significantly reduce the risk of MI [189,190]. These findings suggest that abnormalities in tryptophan metabolism, particularly those related to 5-HT, promote the development of MI comorbid with depression.

In MI combined with depression, no consensus has yet been reached regarding the changes in the tryptophan system or its precise role, from phenotypic alterations to the underlying mechanisms. Clinical observations have shown that plasma tryptophan levels are negatively correlated with IHD severity and that its metabolites (e.g., 5-HT and KYN) are positively correlated with IHD severity. However, Mendelian randomization analyses have found no causal link between these three metabolites and risk factors for IHD (such as diabetes, lipids, or BP) or depression [191]. Thus, tryptophan metabolism may not be the primary driver of IHD or depression; rather, its dysregulation may be a secondary consequence of these conditions. If this is true, studying tryptophan metabolism as a key mechanism in MI comorbid with depression may not be appropriate, and further research is required. Moreover, as peripheral and central 5-HT are largely unable to cross the BBB [192], it may be more effective to study the effects of 5-HT metabolism on depression and cardiovascular health separately: peripheral 5-HT for CVD and central 5-HT for depression. However, BBB disruption in MI combined with depression may enhance communication between the peripheral and central 5-HT systems, which warrants further investigation.

**Figure 4:**
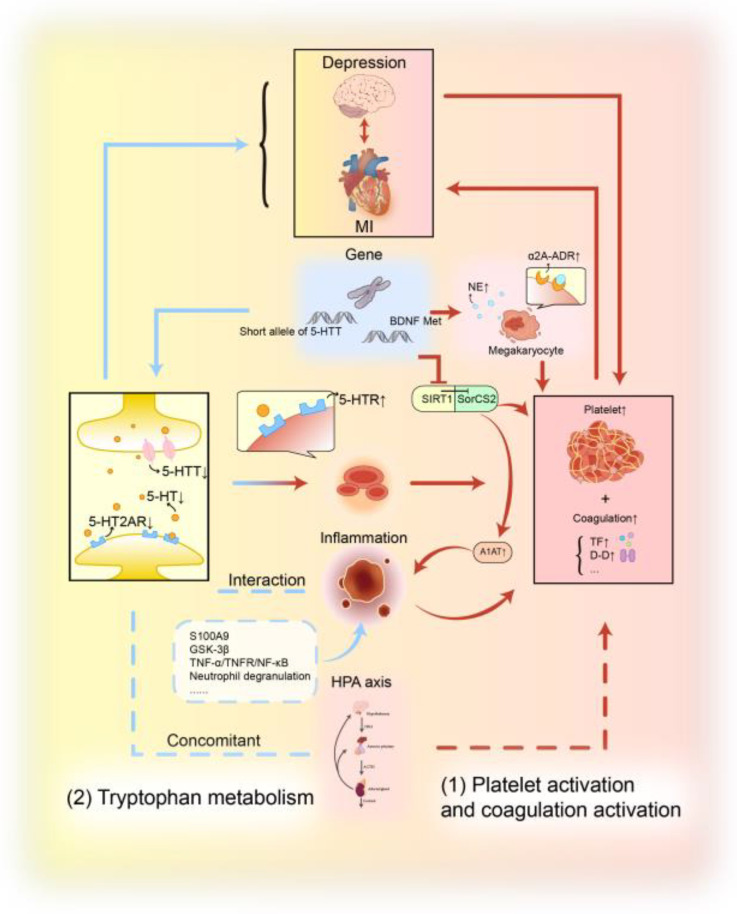
Hyperactivation of platelets and the coagulation system, and tryptophan metabolic disturbances are important mechanisms underlying MI combined with depression. (1) Depression may worsen MI prognosis by promoting thrombosis. The *BDNF Val66Met* polymorphism is a key genetic factor. The *Met variant* enhances coagulation and inflammation via the SIRT1/SorCS2 pathway, and modulates the depression-thrombosis-MI relationship through the NE/α2A-ADR pathway. Reduced 5-HT in depression upregulates platelet 5-HT receptors, increasing platelet activity and thrombotic risk. This hypercoagulable state may also be influenced by HPA axis dysregulation. (2) In MI combined with depression, disrupted tryptophan metabolism usually coincides with HPA axis dysfunction and interacts with inflammation. Mechanisms such as S100A9, GSK-3β, the TNF-α/TNFR/NF-κB signalling pathway, and neutrophil degranulation may mediate inflammation associated with tryptophan metabolism. The *5-HTT short allele* is a genetic risk, potentially disrupting tryptophan metabolism and promoting inflammation. Modulating tryptophan metabolism can simultaneously ameliorate both MI and depression. MI, myocardial infarction; BDNF: brain-derived neurotrophic factor; SIRT1, sirtuin 1; SorCS2, sortilin related VPS10 domain containing receptor 2; NE, norepinephrine; α2A-ADR, α2A-adrenergic receptor; 5-HT, 5-hydroxytryptamine; HPA, hypothalamic–pituitary–adrenal; S100A9, s100 calcium binding protein A9; GSK-3β, glycogen synthase kinase 3 beta; TNF-α, tumour necrosis factor-α; TNFR, TNF-α Receptor; NF-κB, nuclear factor kappa B; TF: tissue factor; D-D: D-dimer; 5-HTT: 5-HT Transporter.

The mechanisms for the Platelet activation and coagulation activation and Tryptophan metabolism are summarised in Figure 4.

### 3.6. Other Mechanisms

#### 3.6.1. Renin–Angiotensin–Aldosterone System

The renin–angiotensin–aldosterone system (RAAS) plays a crucial role in CVD. Angiotensin-converting enzyme 2 (ACE2), a homologue of ACE and an important component of RAAS, degrades angiotensin II (Ang II) into Ang-(1-7). Patients with CAD carrying the ACE2 risk allele demonstrate higher depression scores and reduced Ang II degradation. Depression scores in these patients are negatively correlated with Ang-(1-7) levels and positively correlated with the Ang II/Ang-(1-7) ratio [193]. This suggests that ACE2 genetic polymorphisms may impair ACE2 activity, leading to an Ang II/Ang-(1-7) imbalance, which promotes depression. Angiotensin-converting enzyme inhibitors (ACEIs) and angiotensin receptor blockers are standard therapies for secondary prevention in patients with MI. However, depression reduces patient adherence to these medications by 22%, potentially increasing the risk of adverse cardiovascular outcomes [194].

#### 3.6.2. Endothelial Dysfunction

The vascular endothelium, a single layer of endothelial cells covering the blood vessels, is a critical barrier between peripheral tissues and the circulatory system that regulates blood flow, vascular elasticity, platelet function, and homeostasis. Acute mental stress in patients with stable CAD leads to a 23% reduction in vasodilatory capacity and transient endothelial dysfunction. This transient endothelial dysfunction induced by psychiatric stimuli has been linked to a 78% increased risk of MACE over a 3-year follow-up [195]. Additionally, major depressive episodes significantly reduce levels of circulating endothelial progenitor cells (EPCs) in patients with ACS, compromising post-ACS vascular repair [196]. Moreover, in patients with ACS, endothelin-1 (ET-1) levels also increase with depression, particularly recurrent depression [197]. However, ET-1 levels do not predict MACE risk in young patients with ACS over 2 years [198,199]. Whether this is affected by age remains unknown.

#### 3.6.3. MicroRNAs

MicroRNAs (miRNAs) have emerged as key regulators of the bidirectional relationship between MI and depression and represent a novel area of research. The *miR-146a rs2910164 C allele* reduces miR-146a levels [200], which are correlated with decreased depression risk in patients with CAD [201]. miR-146a may directly target nitric oxide synthase 1 (NOS1) mRNA, thereby affecting its expression [201]. NOS1 plays a crucial role in synaptic signalling and specifically regulates the HPA and 5-HT pathways. Reduced NOS1 expression and activity have been observed in the brains of depressed rats [202,203,204,205]. Thus, the *miR-146a rs2910164 C allele* may upregulate NOS1 expression by reducing miR-146a levels in patients with CAD, thereby potentially mitigating depression. However, because of the limited number of studies on MI-depression comorbidities, the roles of miRNAs such as miR-34a and miR-16 in combined conditions still need further verification.

#### 3.6.4. Gut Microbiome

The gut microbiome has become a focal point for understanding the mechanisms underlying the comorbidity of MI and depression. In CAD, anxiety, and depression, four types of jointly upregulated bacteria (*Staphylococcus*, *Escherichia coli*, *Helicobacter pylori*, and *Shigella*) frequently lead to increased inflammatory response. In contrast, five types of jointly downregulated bacteria (*Prevotella*, *Lactobacillus*, *Faecalibacterium prausnitzii*, *Collinsella*, and *Bifidobacterium*) are usually associated with metabolic abnormalities in short-chain fatty acids, bile acids, and branched-chain amino acids [206]. MI rat models show distinct differences in gut microbiome beta diversity between depressed, non-depressed, and sham groups, with enrichment of *Streptococcus* and a lower abundance of *Actinobacteriaceae* in the depressed group. Notably, the higher area under the curve values for *Streptococcaceae* and *Aspergillus* suggest a predictive link with depression in MI rats. Transplanting the gut flora from depressed rats into antibiotic-treated pseudo-germ-free rats induces depression [207]. Treatment with sotagliflozin and faecal microbiota transplantation (FMT) from sotagliflozin-treated mice can both improve cardiac function and depression [208]. Probiotic Probio-M8 improves Seattle Angina Questionnaire scores and anxiety-depressed symptoms in patients with CAD, reduces serum IL-6 and low-density lipoprotein cholesterol levels, alters gut microbiota composition, and highlights changes in microbial bioactive metabolites, including increased methyl xanthine and decreased pro-atherogenic compounds [209]. Additionally, a combination of *Lactobacillus rhamnosus* and prebiotic inulin is more effective than probiotics alone in reducing depression and inflammation in patients with CAD, likely due to the synergistic effects of prebiotics supporting probiotic growth [210]. Collectively, these findings suggest that the gut–heart–brain axis plays a significant role in the pathology of MI combined with depression, with the gut microbiota representing a potential therapeutic target. However, comprehensive two-sample Mendelian randomization analyses have reported no causal link between gut flora metabolites, colony abundance, depression, CAD, or cardiometabolic markers (e.g., BP, glucose, cholesterol, and body mass index [BMI]) [211]. Given the relatively limited exploration of the gut–heart–brain axis in comorbidities, further research is warranted.

The above mechanisms are summarised in Table 1.

## 4. Interventions and Treatments

Effective interventions for MI combined with depression require careful balancing to optimise outcomes for both cardiac function and mental health. Ideally, treatment strategies should simultaneously support cardiac recovery and mental well-being. In therapies specifically targeting either MI or depression, the potential adverse effects on other conditions must be minimised. Both pharmacological and non-pharmacological approaches can effectively manage this comorbidity.

### 4.1. Interventions

Nonpharmacological interventions, which are often milder in approach, are particularly suitable for managing mild-to-moderate depression and aiding post-MI rehabilitation. Common treatment strategies include psychotherapy and exercise.

#### 4.1.1. Psychotherapy

Recent psychological therapies shown to benefit patients with comorbid MI and depression include cognitive behavioural therapy (CBT), psychocardiology interventions, patient education, comprehensive nursing interventions based on self-expression, and attentional training. These therapies reduce depression and anxiety, enhance sleep quality and life satisfaction, promote physical activity, improve cardiac function, and lower the risk of cardiovascular events [214,215,216,217,218,219,220].

CBT is a widely used modality. Internet-based CBT (iCBT) is particularly effective in improving depressive symptoms in patients with CVD, with benefits sustained at follow-up [218]. Cognitive-behavioural stress management has also shown greater efficacy in reducing depression in patients compared with standard care [219]. In patients with CVD or individuals at risk for CVD, Internet-based self-directed CBT and mindfulness-based cognitive therapy enhance daily physical activity, with increased average step counts during an 8-week follow-up period, whereas participants using only a Fitbit tracker showed reduced physical activity [214]. As increased physical activity benefits cardiac health, CBT may lower CVD risk by encouraging greater physical movement.

In addition to iCBT, emerging digital health tools such as e-health systems, m-health platforms, and telemedicine show promise for managing MI combined with depression. For example, the NEVERMIND e-Health system significantly reduces depression in MI patients [221]. Additionally, a meta-analysis suggested that e-health interventions not only benefit depression but also improve physical health after cardiac surgery [222]. The mobile app AfterAMI improved the quality of life (QoL) of patients with MI [223]. Telemedicine can be used to effectively control BMI and BP, and enhances exercise adherence in patients with atherosclerotic cardiovascular disease, although its effects on mental health and safety outcomes are limited [224]. Overall, Internet-based healthcare has significant potential for the management of MI combined with depression.

#### 4.1.2. Exercise Therapy

Exercise therapy is a viable intervention for improving cardiovascular and mental health. A clinical trial involving 1,282,160 patients with depression observed a 15% reduction in MI risk among individuals who initiated exercise after diagnosis, compared with those who did not exercise. Conversely, patients who stopped exercising had a 15% increased risk compared with those who exercised regularly [225]. Moreover, 12-week high-intensity interval training, moderate-to-vigorous intensity continuous training, and Nordic walking (NW) improve patient cardiac function, depressive symptoms, and QoL, with NW showing a notable advantage in enhancing cardiac function. The benefits of all three exercise regimens persisted for up to 26 weeks [226,227]. These findings highlight the positive effects of regular physical activity on heart health and mood. Additionally, traditional practices such as Tai Chi and Baduanjin may offer similar benefits and potentially enhance the cardiac and psychological improvements of conventional exercise therapy [228,229]. Yoga, which is widely used in rehabilitation, better regulates BP, lipid levels, and psychosocial outcomes (including QoL, stress, anxiety, and depression) in patients with heart disease compared with standard care or non-pharmacological treatments [230]. However, when combined with conventional therapies, yoga may not provide additional benefits [231]. Therefore, yoga may be particularly suitable for patients with MI with mild depression, offering effective relief from cardiac and psychological symptoms without the need for medication.

### 4.2. Medication

Pharmacological treatment of MI combined with depression focuses on three key areas: (1) the impact of standard cardiovascular medications on depression, (2) the effect of antidepressants on the cardiovascular system, and (3) the potential of drugs that address both MI and depression. The following sections discuss both chemical and natural therapeutic options.

#### 4.2.1. Chemical Drugs

Conventional cardiovascular medications are also associated with depression. A meta-analysis revealed that statins and aspirin reduced the risk of depression in patients with CAD by 23% and 15%, respectively. In contrast, the use of calcium channel blockers and diuretics was associated with 32% and 36% increased risks of depression, while β-blockers showed no significant correlation with depression [232]. However, a longitudinal cohort study of 1,400,766 Swedish individuals reported an 8% reduction in hospitalisation for depression with β-blockers but an 8% increase in suicide risk, highlighting a more complex relationship that requires further investigation [233]. Additionally, a prospective randomised controlled trial (RCT) demonstrated that β-blockers increase depressive symptoms in MI patients with preserved LVEF, especially among those with prior β-blockers treatment [234]. Statins not only lower blood lipids and regulate metabolism but are also associated with a reduced risk of depression. The safety and efficacy of combining statins with antidepressants such as SSRIs and serotonin-norepinephrine reuptake inhibitors (SNRIs) have been validated [62].

Common antidepressants, including SSRIs, SNRIs, and tricyclic antidepressants (TCAs), have notable effects on the cardiovascular system. In recent years, SSRIs have garnered significant attention in research on MI combined with depression and 5-HT metabolism. The HUNT study found that SSRIs reduced the risk of MI by 55% [190], whereas a meta-analysis reported that SSRIs were associated with a 44% reduction in MI risk in patients with post-ACS depression [189]. However, conflicting evidence suggests that SSRIs may increase the risk of MACE [235] and the likelihood of CVD-related rehospitalisation by 25% in older adults with a history of CVD [236]. For instance, although escitalopram (ES) significantly improves depression-like behaviours in mice with comorbid MI and depression, it exacerbates cardiac fibrosis [237]. In contrast, sertraline has a relatively high safety profile [238]. The effects of ES on mood may involve inflammatory pathways, as it reverses TNF-α elevation in the cortex of mice with comorbid MI and depression [237]. Overall, the positive and negative cardiovascular effects of SSRIs underscore the complex relationship between 5-HT metabolism and CVD, positioning them as key candidates for the treatment of MI combined with depression.

The risk of MACE associated with SNRIs may be comparable to that associated with SSRIs [239]. Venlafaxine (an SNRI) does not significantly increase the risk of arrhythmias, except in high-risk patients [240]. In the HUNT study involving 317,765 participants, TCAs were associated with a reduced risk of MI [190]. However, a nested case–control study of 344,747 patients found that TCAs may increase the risk of heart failure [236]. Analysis of South Korea’s national claims data revealed that among patients treated with antidepressants, those using TCAs faced the highest risk of atherosclerotic cardiovascular disease when compared with healthy controls [241]. Thus, the relationship between different classes of antidepressants and MI as well as the interaction between standard CVD medications and depression remains controversial. Therefore, careful consideration is necessary when prescribing these medications to patients with comorbid MI and depression.

In addition to conventional CVD medications and antidepressants, several studies have investigated agents that can simultaneously improve MI and depression. Minocycline alleviates cardiac dysfunction and depressive symptoms in post-MI rats by inhibiting inflammation and microglial activation [128]. Probiotics, either alone or in combination with prebiotics, have demonstrated similar effects, likely through mechanisms involving the brain-heart-gut axis and inflammation, although the efficacy may vary by sex [209,210,242]. Emerging research underscores the importance of the brain-heart-gut axis, with sotagliflozin treatment and subsequent FMT to improve both cardiac and psychological outcomes in MI models, primarily by modulating the gut microbiota [208]. These findings suggest that targeting gut flora holds significant promise for the treatment of MI combined with depression.

#### 4.2.2. Natural Compounds

Natural compounds may offer safer and more effective alternatives to chemical drugs because of their multi-target mechanisms and complex bioactive profiles. Network pharmacology analyses have identified quercetin, kaempferol, luteolin, beta-sitosterol, puerarin, stigmasterol, isorhamnetin, baicalein, tanshinone IIa, and nobiletin as key compounds for treating CAD combined with depression, primarily through anti-damage/apoptosis, anti-inflammatory, antioxidative, and neurotransmitter homeostasis functions [243]. The efficacy of formononetin, rosmarinic acid, and extracts from *Hypericum perforatum*, *Ginkgo biloba*, and *Morus macroura* has also been demonstrated in improving the comorbidity [129,133,152,174,212,213]. These findings offer valuable insights for developing novel pharmacological therapies.

Formononetin reduces cardiac injury and depression in MI-depressed mice by inhibiting GSK-3β-mediated Notch1 and C/EBPα signalling pathways, rebalancing the macrophage/microglia M1/M2 ratio and mitigating inflammation [129]. Self-nanoemulsifying self-nanosuspension loaded with *Hypericum perforatum* not only reduces the levels of the myocardial injury markers, NO and TNF-α, improves cardiac function, and restores normal cardiac structure in MI rats but also reduces TNF-α levels in brain tissue, increases neurotransmitter and BDNF levels, upregulates GFAP expression in the cortex and hippocampus, and downregulates BCL2 associated X, apoptosis regulator expression, ultimately alleviating anxiety, depression-like behaviours and cognitive dysfunction [213]. *Ginkgo biloba* extract alleviates depressive-like behaviour and reduces cardiac inflammation and cerebral oxidative stress in rats fed a high-fat diet and with depression by inhibiting the NF-κB signalling pathway [152]. Ginkgolide B improves depression by modulating STAT3-mediated inflammation, inhibiting microglial activation and reducing IL-1β levels in brain tissues of MI mice [133]. The dichloromethane fractions of *Morus macroura* modulate neuronal energy, superoxide dismutase, glutathione, gamma-aminobutyric acid, 5-HT, and dopamine levels, alleviating anxiety and depression in MI rats [174]. In a chronic unpredictable mild stress -induced depression model, prophylactic administration of rosmarinic acid reduced adrenocortical hyperplasia; corticosterone levels; lipid peroxidation; and cTn-I, MMP-2, and pro-inflammatory marker levels while increasing brain 5-HT and glutathione levels, as well as catalase activity, thereby mitigating depression-related cardiac dysfunction through mechanisms involving the HPA axis, oxidative stress, inflammation, and tryptophan metabolism [212]. However, a RCT demonstrated that sustained quercetin supplementation failed to affect endothelial dysfunction biomarkers and depression in post-MI patients [244].

To manage MI combined with depression, non-pharmacological interventions are generally recommended for mild depression, given the incomplete understanding of how antidepressants affect the cardiovascular system. This approach minimises potential cardiovascular harm. When pharmacological treatment is necessary due to severe depression, the use of antidepressants with favourable safety profiles and no interaction with CVD medications is advised. The choice of intervention should be tailored to the severity of MI and depression, emphasising the need for an integrated psychosomatic approach. This necessitates a standardised framework for MI combined with depression that includes recognition, screening, and active management, all guided by integrated and individualised treatment protocols [5]. Cardiologists and mental health specialists should collaborate closely to ensure comprehensive care. Further research is crucial for developing clear clinical guidelines for managing this complex condition.

## 5. Conclusions

The emerging interdisciplinary field of psychocardiology offers significant potential for advancing research and clinical practice. Multiple risk factors substantially increase the risk and worsen the prognosis of MI with comorbid depression, with genetic predisposition further contributing to susceptibility. Complex mechanisms underlie the bidirectional relationship between MI and depression, perpetuating a vicious cycle through the heart–brain axis. Current therapeutic strategies, including non-pharmacological interventions such as psychotherapy and exercise, as well as pharmacological and natural medications, can effectively manage this comorbidity. However, existing research has disproportionately focused on factors such as sex and diabetes, whereas evidence for other contributors remains scarce or controversial. Moreover, the generalizability of the research findings may be limited by cross-cultural differences in the perception of stress. This underscores the need for large-scale, multicentre, prospective trials to clarify the risk factors and outcomes. Mechanistic studies should extend beyond phenotypic associations to investigate upstream and downstream molecular pathways. The insights from these studies can guide the development of more precise, safe, and effective treatments. The identification of risk factors and pathogenic mechanisms provides a foundation for leveraging advanced tools such as artificial intelligence-driven data phenotyping, machine learning, and predictive model construction to enhance diagnosis and treatment strategies. Bioinformatic approaches, including proteomics, may uncover specific biomarkers for MI with depression, enabling early detection, accurate diagnosis, and personalised therapy. The current reliance on subjective scales to assess depression highlights the need for more objective diagnostic methods. Psychosomatic symptoms such as chest tightness and chest pain, which can reflect underlying depression, are often overlooked in clinical practice, leading to misdiagnosis, inefficient resource use, and potential iatrogenic harm. Interdisciplinary collaboration is crucial for the development of an integrated diagnostic and treatment framework. Continued physician education must be prioritised along with public health initiatives to raise awareness of mental health issues, improve patient understanding, and reduce stigma. These efforts will advance the comprehensive prevention, diagnosis, and management of MI and depression, foster better patient outcomes, and optimise healthcare resources.

## Figures and Tables

**Table 1:** Summary of phenotypes, mechanisms and interventions.

	Effect of MI on Depression			Effects of Depression on MI		
	Phenotypes	Mechanisms	Interventions	Phenotypes	Mechanisms	Interventions
ANS	HRV ↓ [79]	GRK2 ↑→ HRV ↓, depression and MI [83,84,85,86]	Paroxetine [86]	HRV ↓, plasma epinephrine and NE levels ↑ [87,88,89,90]	S1R↓ (depression-related) → ANS dysfunction → Cardiac function and structure ↓ [91]	
HPA axis	GC ↑, adrenal hypertrophy and flattened DCS [97,98,99]	MI→ Inflammation (mRNA expression of IL-12A and TNF-α in the hypothalamus ↑) → HPA axis dysregulation → Depression [102]. MI → Trigger HPA axis dysfunction after MR/GR imbalance → Depression [100] Impaired CR signalling [100,103,108,110]		GC ↑ and flattened DCS [104,105,106,107]	Depression → Abnormal activation of the HPA axis → GR-mediated NF-κB/TLR4 signalling pathway ↑ → CVD [108,109] Impaired CR signalling [100,103,108,110]	Rosmarinic acid [212]
Inflammation	Hs-CRP, NLRP3, TNF-α, IL-17A, IL-1β, IL-2, IL-6, NLR and microglia ↑ [123,124,125,126,127,128]	GSK-3β/Notch1 and GSK-3β/C/EBPα signalling pathways induce macrophage/microglial polarisation [129] NLRP3-mediated GSDMD-induced microglial pyroptosis [130] S100A9-mediated macrophage/microglial inflammation [131,132] S100B, JMJD3 or STAT3-mediated microglial polarization [133,134,135,136,137]	Minocycline [128] Formononetin [129] HP.SNESNS [213] GBE [152] Ginkgolide B [133]	Occurrence: SII and hs-CRP [58,138] Prognosis: MIF and CRP [140,141,142,143]	Depression → 5-HTT ↓ →inflammation (TGF-β ↑, TNF-α ↑, IL-6 ↑ and MMP-2 ↑) → The early healing in MI ↓ [144,145,146,147] Depression → PG/GAG structural and functional changes → Heart disease [148,149,150]	Formononetin [129] Rosmarinic acid [212]
Platelet activation and coagulation activation				Platelet activation: the average PLT, PDW and MPV ↑ [155]. Coagulation activation: TF and D-dimer ↑ [156,157]	Depression → 5-HT ↓ → Platelet activation → CVD ↑ [158,159,160] The *BDNF Val66Met gene* polymorphism: *BDNF ^Met^* → SIRT1 ↓/SorCS2 ↑ pathway → regulates coagulation (TF ↑ and gelsolin ↑) and inflammation (A1AT ↑), promotes thrombosis and increases CVD risk [162] *BDNF ^Met^*→ NE/α_2A_-ADR pathway ↑ → Thrombosis → CVD risk ↑ [165]	Sertraline and N-nitrosodimethylamine [160] Desipramine [165] Rauwolscine [165]
RAAS	Ang-(1-7) ↓ Ang-Ⅱ/Ang-(1-7) ↑ [193]	ACE2 genetic polymorphisms: ACE2 genetic polymorphisms in patients with CHD → ACE2 ↓ → Ang II/Ang-(1-7) imbalance → Depression [193]			Depression reduces adherence to ACEIs in patients with MI [194].	
Endothelial dysfunction				Vasodilatory capacity ↓, EPC ↓ and ET-1 ↑ [195,196,197].		
MiRNA		*MiR-146a rs2910164 C allele* → MiR-146a ↓ (patients with CAD) → NOS1 ↑ → Depression ↓ [200,201,202,203,204,205]				
	Phenotypes		Mechanisms			Interventions
Tryptophan metabolism	5-HT levels in serum and brain tissue ↓ [131,173,174,175] 5-HT2A receptors expression in brain tissue ↓ [175,176] Altered plasma-free L-Trp scores alongside abnormal IDAEP [177] The mRNA expression of tryptophan metabolism-related enzymes such as IDO1, KMO and KYNU in the hippocampus↑ [173] Serum KYN levels ↑ [134] KYN-to-tryptophan ratio ↑ [134]		HPA axis [134,173] Inflammation (may be mediated by S100A9, GSK-3β, TNF-α/TNFR/NF-κB signalling pathway or neutrophil degranulation) → The inflammation in the infarcted cardiac tissue ↑ and depression ↑ [129,131,134,173,185,186] Regulation of *5-HTT* gene polymorphisms → Depression and 5-HTT ↓ → Inflammation ↑ → Cardiac outcomes ↓ [144,145,147,187] 5-HT ↓ → Thrombosis ↑ → MI risk ↑ [158,159,160,188,189,190]			SSRIs [189,190] Dichloromethane fractions of Morus macroura [174] Rosmarinic acid [212]
Gut microbiome	*Staphylococcus*, *Escherichia coli*, *Helicobacter pylori*, *Shigella* and *Streptococcus* ↑ [206] *Prevotella*, *Lactobacillus*, *E. pumilus*, *Collinsella*, *Bifidobacterium* and *Actinobacteriaceae* ↓ [206] AUC values for *Streptococcaceae* and *Aspergillus* ↑ [207]		The gut–heart–brain axis			Sotagliflozin, [208] probiotic and prebiotic inulin [209,210]

Symbols: ↑: increase in a value or deterioration of a disease; ↓: decrease in a value or improvement of a disease; →: denotes a causative relationship (leads to). Abbreviations: MI: myocardial infarction; ANS: autonomic nervous system; HRV: heart rate variability; GRK2: G protein-coupled receptor kinase-2; NE: norepinephrine; S1R: sigma-1 receptor; HPA: hypothalamic–pituitary–adrenal; ACTH: adrenocorticotropic hormone; GC: glucocorticoid; DCS: diurnal cortisol slope; IL-12: interleukin-12A; TNF-α: tumour necrosis factor-α; MR/GR: mineralocorticoid receptors/glucocorticoid receptors; CR: corticosteroid receptor; NF-κB: nuclear factor kappa B; TLR4: toll-like receptor 4; CVD: cardiovascular disease; CRP: C-reactive protein; Hs-CRP: high-sensitivity CRP; NLRP3: NOD-like receptor thermal protein domain associated protein 3; NLR: neutrophil-to-lymphocyte ratio; GSK-3β: glycogen synthase kinase 3 beta; Notch1, notch receptor 1; C/EBPα: CCAAT/enhancer-binding protein alpha; GSDMD: gasdermin-D; S100A9: s100 calcium binding protein A9; S100B: s100 calcium binding protein B; JMJD3: jumonji domain-containing protein 3; STAT3: signal transducer and activator of transcription 3; HP.SNESNS: Self-nanoemulsifying self-nanosuspension loaded with Hypericum perforatum; GBE: Ginkgo biloba extract; SII: Systemic Immune Inflammation Index; MIF: migration inhibitory factor; 5-HTT: 5-HT transporter; TGF-β: transforming growth factor-β; MMP2: matrix metalloproteinase 2; PG: Proteoglycan; GAG: glycosaminoglycan; PLT: platelet count; PDW: platelet distribution width; MPV: mean platelet volume; TF: tissue factor; BDNF: brain-derived neurotrophic factor; SIRT1: sirtuin 1; SorCS2: sortilin related VPS10 domain containing receptor 2; A1AT: α1-antitrypsin; α2A-ADR: α2A-adrenergic receptor; RAAS: renin–angiotensin–aldosterone system; Ang II: angiotensin II; ACE2: angiotensin-converting enzyme 2; CHD: coronary heart disease; ACEI: Angiotensin-converting enzyme inhibitor; EPC: endothelial progenitor cell; ET-1: endothelin-1; NOS1: nitric oxide synthase 1; L-Trp: L-tryptophan; IDAEP: intensity-dependent auditory evoked potentials; IDO1: indoleamine 2,3-dioxygenase 1; KMO: kynurenine 3-monooxygenase; KYNU: kynureninase; KYN: kynurenine; TNFR: TNF-α Receptor. SSRI: selective serotonin reuptake inhibitor; AUC: area under the curve.

## Data Availability

No new data were created or analysed in this study.

## Abbreviations

The following abbreviations are used in this manuscript:

MI	Myocardial Infarction
ANS	Autonomic Nervous System
HPA	Hypothalamic-pituitary-pdrenal
RAAS	Renin–angiotensin–aldosterone System
MDD	Major Depressive Disorder
CVD	Cardiovascular Disease
MACE	Major Adverse Cardiovascular Events
CAD	Coronary Artery Disease
CHD	Coronary Heart Disease
ACS	Acute Coronary Syndrome
IHD	Ischaemic Heart Disease
LDL	Low-density Lipoprotein
PCSK9	Pro-protein Convertase Subtilisin/kexin Type 9
HRV	Heart Rate Variability
FKBP5	FK506 binding Protein 51
APLNR	Apelin receptor
SNS	Sympathetic Nervous System
PNS	Parasympathetic Nervous System
HR	Heart Rate
NE	Norepinephrine
ACh	Acetylcholine
S1R	Sigma-1 Receptor
GRK2	G Protein-coupled Receptor Kinase-2
LVEF	Left Ventricular Ejection Fractions
BP	Blood Pressure
SSRI	Selective Serotonin Reuptake Inhibitor
LVEDP	Left Ventricular End-diastolic Pressure
I/R	Ischemia/Reperfusion
CRH	Corticotropin-releasing Hormone
ACTH	Adrenocorticotropic Hormone
GC	Glucocorticoid
CRs	Cortisol Receptors
MRs	Mineralocorticoid Receptors
GRs	glucocorticoid receptors
DCS	Diurnal Cortisol Slope
GFAP	Glial Fibrillary Acidic Protein
IL	Interleukin
TNF	Tumour Necrosis Factor
NR3C1	Nuclear Receptor Subfamily 3 Group C Member 1
HDL-C	High-density Lipoprotein Cholesterol
TLR4	Toll-like Receptor 4
NF-κB	Nuclear Factor Kappa B
CR	Corticosteroid Receptor
CAR	Cortisol Awakening Response
BBB	Blood–Brain Barrier
MMPs	Matrix Metalloproteinases
CNS	Central Nervous System
hs-CRP	High-sensitivity CRP
PCI	Percutaneous Coronary Intervention
NLRP3	NOD-like Receptor Thermal Protein Domain Associated Protein 3
STEMI	ST-segment Elevation Myocardial Infarction
NLR	Neutrophil To Lymphocyte Ratio
GSK-3β	Glycogen Synthase Kinase 3 beta
S100A9	S100 Calcium Binding Protein A9
S100B	S100 Calcium Binding Protein B
JMJD3	Jumonji Domain-containing Protein 3
STAT3	Signal Transducer and Activator of Transcription 3
Notch1	Notch Receptor 1
C/EBPα	CCAAT/Enhancer-Binding Protein Alpha
GSDMD	Gasdermin-D
SII	Systemic Immune Inflammation Index
MIR	Recurrent Myocardial Infarction
MIF	Migration Inhibitory Factor
AMPK	Adenosine 5′-monophosphate-activated Protein Kinase
5-HTT/SERT	5-HT Transporter
TGF-β	Transforming Growth Factor-β
PG	Proteoglycan
GAG	Glycosaminoglycan
HS	Heparan Sulphate
CS	Chondroitin Sulphate
MPO	Myeloperoxidase
TF	Tissue Factor
5-HT	5-Hydroxytryptamine
BDNF	Brain-Derived Neurotrophic Factor
SIRT1	Sirtuin 1
SorCS2	Sortilin Related VPS10 Domain Containing Receptor 2
A1AT	α1-Antitrypsin
α2A-ADR	α2A-Adrenergic Receptor
L-Trp	L-tryptophan
KYNU	kynureninas
KYN	kynurenine
TNFR	TNF-α Receptor
ACE2	Angiotensin-Converting Enzyme 2
Ang II	Angiotensin II
ACEI	Angiotensin-Converting Enzyme Inhibitor
EPC	Endothelial Progenitor Cell
ET-1	Endothelin-1
MiRNAs	MicroRNAs
NOS1	Nitric Oxide Synthase 1
FMT	Faecal Microbiota Transplantation
CBT	Cognitive Behavioural Therapy
QoL	Quality of life
NW	Nordic Walking
RCT	Randomised Controlled Trial
SNRIs	Serotonin-norepinephrine Reuptake Inhibitors
TCA	Tricyclic Antidepressants
ES	Escitalopram

## References

[1] GBD 2023 Disease and Injury and Risk Factor Collaborators (2025). Burden of 375 Diseases and Injuries, Risk-Attributable Burden of 88 Risk Factors, and Healthy Life Expectancy in 204 Countries and Territories, Including 660 Subnational Locations, 1990–2023: A Systematic Analysis for the Global Burden of Disease Study 2023. Lancet.

[2] Malhi G.S., Mann J.J. (2018). Depression. Lancet.

[3] Chan J.K.N., Solmi M., Lo H.K.Y., Chan M.W.Y., Choo L.L.T., Lai E.T.H., Wong C.S.M., Correll C.U., Chang W.C. (2025). All-Cause and Cause-Specific Mortality in People with Depression: A Large-Scale Systematic Review and Meta-Analysis of Relative Risk and Aggravating or Attenuating Factors, Including Antidepressant Treatment. World Psychiatry.

[4] Zeng J., Qiu Y., Yang C., Fan X., Zhou X., Zhang C., Zhu S., Long Y., Hashimoto K., Chang L. (2025). Cardiovascular Diseases and Depression: A Meta-Analysis and Mendelian Randomization Analysis. Mol. Psychiatry.

[5] Levine G.N., Carney R.M., Cohen B.E., Dunn S.L., Gaffey A.E., Kronish I.M., Olsson E.M.G., Huffman J.C., American Heart Association Stroke Council, Council on Cardiovascular and Stroke Nursing (2025). Post-Myocardial Infarction Psychological Distress: A Scientific Statement From the American Heart Association. Circulation.

[6] Narendrula A., Ajani K., Lang J., Brinza E., Longenecker C.T. (2023). Psychological Distress and Health Perception in Patients with a Previous Myocardial Infarction or Stroke: A National Cross-Sectional Study. BMC Cardiovasc. Disord..

[7] Feng L., Li L., Liu W., Yang J., Wang Q., Shi L., Luo M. (2019). Prevalence of Depression in Myocardial Infarction: A PRISMA-Compliant Meta-Analysis. Medicine.

[8] Lin Y., Bai W., Liu H.-H., Li Z.-Z., Gao Z.-Z., Han T., Ren H.-H., Ng C.H., Xiang Y.-T. (2023). Prevalence, Correlates, and Network Analysis of Depression and Its Association with Quality of Life in Survivors with Myocardial Infarction during the COVID-19 Pandemic. J. Affect. Disord..

[9] Meijer A., Conradi H.J., Bos E.H., Anselmino M., Carney R.M., Denollet J., Doyle F., Freedland K.E., Grace S.L., Hosseini S.H. (2013). Adjusted Prognostic Association of Depression Following Myocardial Infarction with Mortality and Cardiovascular Events: Individual Patient Data Meta-Analysis. Br. J. Psychiatry.

[10] Sreenivasan J., Kaul R., Khan M.S., Malik A., Usman M.S., Michos E.D. (2022). Mental Health Disorders and Readmissions Following Acute Myocardial Infarction in the United States. Sci. Rep..

[11] Mohamed M.O., Rashid M., Farooq S., Siddiqui N., Parwani P., Shiers D., Thamman R., Gulati M., Shoaib A., Chew-Graham C. (2019). Acute Myocardial Infarction in Severe Mental Illness: Prevalence, Clinical Outcomes, and Process of Care in U.S. Hospitalizations. Can. J. Cardiol..

[12] Lee S.N., Yun J.-S., Ko S.-H., Ahn Y.-B., Yoo K.-D., Her S.-H., Moon D., Jung S.-H., Won H.-H., Kim D. (2023). Impacts of Gender and Lifestyle on the Association between Depressive Symptoms and Cardiovascular Disease Risk in the UK Biobank. Sci. Rep..

[13] Lichtman J.H., Froelicher E.S., Blumenthal J.A., Carney R.M., Doering L.V., Frasure-Smith N., Freedland K.E., Jaffe A.S., Leifheit-Limson E.C., Sheps D.S. (2014). Depression as a Risk Factor for Poor Prognosis among Patients with Acute Coronary Syndrome: Systematic Review and Recommendations: A Scientific Statement from the American Heart Association. Circulation.

[14] Wu S., Yang X., Chen Y., Wang Y., Liang H., Xu W., Wang J., Shao X., Zhang H., Zhong Z. (2025). Changes in Depressive Symptoms as Predictors of Incident Cardiovascular Disease: Insights from Four Prospective Cohorts. Eur. J. Prev. Cardiol..

[15] Walli-Attaei M., Rosengren A., Rangarajan S., Breet Y., Abdul-Razak S., Sharief W.A., Alhabib K.F., Avezum A., Chifamba J., Diaz R. (2022). Metabolic, Behavioural, and Psychosocial Risk Factors and Cardiovascular Disease in Women Compared with Men in 21 High-Income, Middle-Income, and Low-Income Countries: An Analysis of the PURE Study. Lancet.

[16] Kovess-Masfety V., Boyd A., van de Velde S., de Graaf R., Vilagut G., Haro J.M., Florescu S., O’Neill S., Weinberg L., Alonso J. (2014). Are There Gender Differences in Service Use for Mental Disorders across Countries in the European Union? Results from the EU-World Mental Health Survey. J. Epidemiol. Community Health.

[17] Al-Zaru I.M., Alhalaiqa F., Dalky H.F., Arramadan K.A., Batiha A.-M. (2020). Depression in Nonhospitalized Jordanian Patients With Coronary Artery Disease. J. Nurs. Res..

[18] Dikić A., Radmilo L., Živanović Ž., Keković G., Sekulić S., Kovačić Z., Radmilo R. (2021). Cognitive Impairment and Depression after Acute Myocardial Infarction: Associations with Ejection Fraction and Demographic Characteristics. Acta Neurol. Belg..

[19] Zhu C., Tran P., Dreyer R., Lichtman J. (2021). The Association of Depression With Cardiac Rehabilitation Attendance by Age and Sex Among Patients With Myocardial Infarction: Results From the Behavioral Risk Factor Surveillance System. Circulation.

[20] Nyström A., Strömberg S., Jansson K., Faresjö Å.O., Faresjö T. (2022). Cardiovascular Risks before Myocardial Infarction Differences between Men and Women. BMC Cardiovasc. Disord..

[21] Lu Y., Li S.-X., Liu Y., Rodriguez F., Watson K.E., Dreyer R.P., Khera R., Murugiah K., D’Onofrio G., Spatz E.S. (2022). Sex-Specific Risk Factors Associated With First Acute Myocardial Infarction in Young Adults. JAMA Netw. Open.

[22] Moreno G., Vicent L., Rosillo N., Delgado J., Cerro E.P.D., Bueno H. (2024). Do Sex and Gender Aspects Influence Non-Adherence to Secondary Prevention Measures after Myocardial Infarction?. Am. J. Prev. Cardiol..

[23] Theofilis P., Oikonomou E., Lazaros G., Vogiatzi G., Niarchou P., Goliopoulou A., Anastasiou M., Mistakidi V.C., Tsalamandris S., Fountoulakis P. (2023). The Association of Depression With QT Duration: A Comparison Between Individuals Younger or Older Than 65 Years. Psychosom. Med..

[24] Nygren A., Reutfors J., Karlsson P., Tiger M., Faxén J., Brenner P. (2025). Risk of Major Adverse Cardiovascular Events in Treatment-Resistant or Severe Depression. J. Affect. Disord..

[25] Erdman J., Kornspun A., Stein L., Rossi K., Tuhrim S., Dhamoon M. (2020). Readmission for Depression and Suicide Attempt Following Stroke and Myocardial Infarction. Stroke.

[26] Fleetwood K., Wild S.H., Smith D.J., Mercer S.W., Licence K., Sudlow C.L.M., Jackson C.A. (2021). Severe Mental Illness and Mortality and Coronary Revascularisation Following a Myocardial Infarction: A Retrospective Cohort Study. BMC Med..

[27] Lang X., Liu Z., Islam S., Han G., Rangarajan S., Tse L.A., Mushtaha M., Wang J., Hu L., Qiang D. (2022). Interaction of Depression and Unhealthy Diets on the Risk of Cardiovascular Diseases and All-Cause Mortality in the Chinese Population: A PURE Cohort Substudy. Nutrients.

[28] Wang H., Liu F., Ma H., Yin H., Wang P., Bai B., Guo L., Geng Q. (2021). Associations between Depression, Nutrition, and Outcomes among Individuals with Coronary Artery Disease. Nutrition.

[29] Pogosova N., Boytsov S., De Bacquer D., Sokolova O., Ausheva A., Kursakov A., Saner H. (2021). Factors Associated with Anxiety and Depressive Symptoms in 2775 Patients with Arterial Hypertension and Coronary Heart Disease: Results from the COMETA Multicenter Study. Glob. Heart.

[30] Kim M., Kim H., Han K., Yoo J., Yang K., Jeon H.J. (2022). Changes in Alcohol Consumption and the Risk of Cardiovascular Diseases in Patients with Depression Who Had Not Consumed Alcohol: A Nationwide Cohort Study. J. Psychiatr. Res..

[31] Lu Y., Wang Z., Georgakis M.K., Lin H., Zheng L. (2021). Genetic Liability to Depression and Risk of Coronary Artery Disease, Myocardial Infarction, and Other Cardiovascular Outcomes. J. Am. Heart Assoc..

[32] Li G.H.-Y., Cheung C.-L., Chung A.K.-K., Cheung B.M.-Y., Wong I.C.-K., Fok M.L.Y., Au P.C.-M., Sham P.-C. (2022). Evaluation of Bi-Directional Causal Association between Depression and Cardiovascular Diseases: A Mendelian Randomization Study. Psychol. Med..

[33] Zhang W.-Y., Nan N., He Y., Zuo H.-J., Song X.-T., Zhang M., Zhou Y. (2023). Prevalence of Depression and Anxiety Symptoms and Their Associations with Cardiovascular Risk Factors in Coronary Patients. Psychol. Health Med..

[34] Furlong-Millones M.R., Mostacero-Becerra K., Aguirre-Milachay E., Alvarez-Risco A., Del-Aguila-Arcentales S., Garcia Guerra A., Davies N.M., Yañez J.A., Valladares-Garrido M.J. (2022). Quality of Life, Anxiety, and Depression in Peruvian Patients with Acute Coronary Syndrome. Sustainability.

[35] Murphy B., Le Grande M., Alvarenga M., Worcester M., Jackson A. (2019). Anxiety and Depression After a Cardiac Event: Prevalence and Predictors. Front. Psychol..

[36] Krasieva K., Clair C., Gencer B., Carballo D., Klingenberg R., Raber L., Windecker S., Rodondi N., Matter C.M., Luscher T.F. (2021). Impact of Smoking Cessation on Depression after Acute Coronary Syndrome. Eur. Heart J..

[37] Krasieva K., Clair C., Gencer B., Carballo D., Klingenberg R., Räber L., Windecker S., Rodondi N., Matter C.M., Lüscher T.F. (2022). Smoking Cessation and Depression after Acute Coronary Syndrome. Prev. Med..

[38] Deschênes S.S., Burns R.J., Graham E., Schmitz N. (2019). Depressive Symptoms and Sleep Problems as Risk Factors for Heart Disease: A Prospective Community Study. Epidemiol. Psychiatr. Sci..

[39] Cheng X., Ouyang F., Ma T., He L., Gong L., Yin J., Zhang G., Bai Y. (2022). The Individual and Joint Associations of Depression and Sleep Duration with Cardiometabolic Diseases and Mortality: A Prospective Cohort Study. Atherosclerosis.

[40] Feng Z., Tong W.K., Tang Z. (2022). Longitudinal Trends in the Prevalence and Treatment of Depression among Adults with Cardiovascular Disease: An Analysis of National Health and Nutrition Examination Survey 2009–2020. Front. Psychiatry.

[41] Lee C., Lee S.C., Shin Y.S., Park S., Won K.B., Ann S.H., Ko E.J. (2020). Severity, Progress, and Related Factors of Mood Disorders in Patients with Coronary Artery Disease: A Retrospective Study. Healthcare.

[42] Park S.J., Lee M.G., Jo M., Kim G., Park S. (2020). Joint Effect of Depression and Health Behaviors or Conditions on Incident Cardiovascular Diseases: A Korean Population-Based Cohort Study. J. Affect. Disord..

[43] Rajan S., McKee M., Rangarajan S., Bangdiwala S., Rosengren A., Gupta R., Kutty V.R., Wielgosz A., Lear S., AlHabib K.F. (2020). Association of Symptoms of Depression With Cardiovascular Disease and Mortality in Low-, Middle-, and High-Income Countries. JAMA Psychiatry.

[44] Silventoinen K., Korhonen K., Lahtinen H., Jelenkovic A., Havulinna A.S., Ripatti S., Salomaa V., Davey Smith G., Martikainen P. (2022). Joint Associations of Depression, Genetic Susceptibility and the Area of Residence for Coronary Heart Disease Incidence. J. Epidemiol. Community Health.

[45] Power N., Deschênes S.S., Ferri F., Schmitz N. (2020). The Association between Job Strain, Depressive Symptoms, and Cardiovascular Disease Risk: Results from a Cross-Sectional Population-Based Study in Québec, Canada. Int. Arch. Occup. Environ. Health.

[46] Jones D.P., Wootton R.E., Gill D., Carter A.R., Gunnell D., Munafò M.R., Sallis H.M. (2021). Mental Health as a Mediator of the Association Between Educational Inequality and Cardiovascular Disease: A Mendelian Randomization Study. J. Am. Heart Assoc..

[47] Figura A., Kuhlmann S.L., Rose M., Slagman A., Schenk L., Möckel M. (2021). Mental Health Conditions in Older Multimorbid Patients Presenting to the Emergency Department for Acute Cardiac Symptoms: Cross-Sectional Findings from the EMASPOT Study. Acad. Emerg. Med..

[48] Niedhammer I., Sultan-Taïeb H., Parent-Thirion A., Chastang J.-F. (2022). Update of the Fractions of Cardiovascular Diseases and Mental Disorders Attributable to Psychosocial Work Factors in Europe. Int. Arch. Occup. Environ. Health.

[49] Munyombwe T., Dondo T.B., Aktaa S., Wilkinson C., Hall M., Hurdus B., Oliver G., West R.M., Hall A.S., Gale C.P. (2021). Association of Multimorbidity and Changes in Health-Related Quality of Life Following Myocardial Infarction: A UK Multicentre Longitudinal Patient-Reported Outcomes Study. BMC Med..

[50] Jia Z., Li S. (2021). Risk of Cardiovascular Disease Mortality in Relation to Depression and 14 Common Risk Factors. Int. J. Gen. Med..

[51] Inoue K., Mayeda E.R., Nianogo R., Paul K., Yu Y., Haan M., Ritz B. (2021). Estimating the Joint Effect of Diabetes and Subsequent Depressive Symptoms on Mortality among Older Latinos. Ann. Epidemiol..

[52] Jung I., Kwon H., Park S.E., Han K.-D., Park Y.-G., Kim Y.-H., Rhee E.-J., Lee W.-Y. (2021). Increased Risk of Cardiovascular Disease and Mortality in Patients with Diabetes and Coexisting Depression: A Nationwide Population-Based Cohort Study. Diabetes Metab. J..

[53] Peter R.S., Jaensch A., Mons U., Schöttker B., Schmucker R., Koenig W., Brenner H., Rothenbacher D. (2021). Prognostic Value of Long-Term Trajectories of Depression for Incident Diabetes Mellitus in Patients with Stable Coronary Heart Disease. Cardiovasc. Diabetol..

[54] Zareini B., Sørensen K.K., Blanche P., Falkentoft A.C., Fosbøl E., Køber L., Torp-Pedersen C. (2024). Incidence of Depression in Patients with Cardiovascular Disease and Type 2 Diabetes: A Nationwide Cohort Study. Clin. Res. Cardiol..

[55] Li W., Yin H., Liu Q., Chen Y., Liang Y., Zhou H., Ma H., Geng Q. (2022). Associations Among Depression, Hemoglobin A1c Level, and Prognosis in Patients With Coronary Artery Disease: A Prospective Study. Front. Psychiatry.

[56] Chen X., Liu Z., Yang Y., Chen G., Wan Q., Qin G., Yan L., Wang G., Qin Y., Luo Z. (2022). Depression Status, Lifestyle, and Metabolic Factors With Subsequent Risk for Major Cardiovascular Events: The China Cardiometabolic Disease and Cancer Cohort (4C) Study. Front. Cardiovasc. Med..

[57] Huang P., Yan L., Li Z., Zhao S., Feng Y., Zeng J., Chen L., Huang A., Chen Y., Lei S. (2023). Potential Shared Gene Signatures and Molecular Mechanisms between Atherosclerosis and Depression: Evidence from Transcriptome Data. Comput. Biol. Med..

[58] Lin J., Yang R., Zhang Y., Hou Y., Yang H., Zhou X., Liu T., Yang Q., Wang Y. (2023). The Mediation Effects of Metabolic and Immune-Inflammation Factors on the Depression-Premature Coronary Heart Disease Association. J. Affect. Disord..

[59] Katzmann J.L., Mahfoud F., Böhm M., Schulz M., Laufs U. (2019). Association of Medication Adherence and Depression with the Control of Low-Density Lipoprotein Cholesterol and Blood Pressure in Patients at High Cardiovascular Risk. Patient Prefer. Adherence.

[60] Macchi C., Favero C., Ceresa A., Vigna L., Conti D.M., Pesatori A.C., Racagni G., Corsini A., Ferri N., Sirtori C.R. (2020). Depression and Cardiovascular Risk-Association among Beck Depression Inventory, PCSK9 Levels and Insulin Resistance. Cardiovasc. Diabetol..

[61] Akyol O., Chowdhury I., Akyol H.R., Tessier K., Vural H., Akyol S. (2020). Why Are Cardiovascular Diseases More Common among Patients with Severe Mental Illness? The Potential Involvement of Electronegative Low-Density Lipoprotein (LDL) L5. Med. Hypotheses.

[62] Gutlapalli S.D., Farhat H., Irfan H., Muthiah K., Pallipamu N., Taheri S., Thiagaraj S.S., Shukla T.S., Giva S., Penumetcha S.S. (2022). The Anti-Depressant Effects of Statins in Patients With Major Depression Post-Myocardial Infarction: An Updated Review 2022. Cureus.

[63] Khandaker G.M., Zuber V., Rees J.M.B., Carvalho L., Mason A.M., Foley C.N., Gkatzionis A., Jones P.B., Burgess S. (2021). Correction: Shared Mechanisms between Coronary Heart Disease and Depression: Findings from a Large UK General Population-Based Cohort. Mol. Psychiatry.

[64] Shell A.L., Gonzenbach V., Sawhney M., Crawford C.A., Stewart J.C. (2022). Associations between Affective Factors and High-Frequency Heart Rate Variability in Primary Care Patients with Depression. J. Psychosom. Res..

[65] Yıldırım D., Kocatepe V. (2023). Evaluating Death Anxiety and Death Depression Levels among Patients with Acute Myocardial Infarction. Omega.

[66] Bobo W.V., Grossardt B.R., Virani S., St Sauver J.L., Boyd C.M., Rocca W.A. (2022). Association of Depression and Anxiety With the Accumulation of Chronic Conditions. JAMA Netw. Open.

[67] Wang Q., Shelton R.C., Dwivedi Y. (2018). Interaction between Early-Life Stress and FKBP5 Gene Variants in Major Depressive Disorder and Post-Traumatic Stress Disorder: A Systematic Review and Meta-Analysis. J. Affect. Disord..

[68] Brandt J., Warnke K., Jörgens S., Arolt V., Beer K., Domschke K., Haverkamp W., Kuhlmann S.L., Müller-Nordhorn J., Rieckmann N. (2020). Association of FKBP5 Genotype with Depressive Symptoms in Patients with Coronary Heart Disease: A Prospective Study. J. Neural Transm..

[69] Wang P., Xu C., Wang C., Wu Y., Wang D., Chen S., Zhao Y., Wang X., Li S., Yang Q. (2015). Association of SNP Rs9943582 in APLNR with Left Ventricle Systolic Dysfunction in Patients with Coronary Artery Disease in a Chinese Han GeneID Population. PLoS ONE.

[70] Chen T., Wu B., Lin R. (2017). Association of Apelin and Apelin Receptor with the Risk of Coronary Artery Disease: A Meta-Analysis of Observational Studies. Oncotarget.

[71] Wang Y., Liu W., Xiao Y., Yuan H., Wang F., Jiang P., Luo Z. (2020). Association of Apelin and Apelin Receptor Polymorphisms With the Risk of Comorbid Depression and Anxiety in Coronary Heart Disease Patients. Front. Genet..

[72] Sun Y., Zhang H., Wang B., Chen C., Chen Y., Chen Y., Xia F., Tan X., Zhang J., Li Q. (2022). Joint Exposure to Positive Affect, Life Satisfaction, Broad Depression, and Neuroticism and Risk of Cardiovascular Diseases: A Prospective Cohort Study. Atherosclerosis.

[73] Aires R., Pimentel E.B., Forechi L., Dantas E.M., Mill J.G. (2017). Time Course of Changes in Heart Rate and Blood Pressure Variability in Rats with Myocardial Infarction. Braz. J. Med. Biol. Res..

[74] Mulkey S.B., du Plessis A.J. (2019). Autonomic Nervous System Development and Its Impact on Neuropsychiatric Outcome. Pediatr. Res..

[75] Pyhälä R., Wolford E., Kautiainen H., Andersson S., Bartmann P., Baumann N., Brubakk A.-M., Evensen K.A.I., Hovi P., Kajantie E. (2017). Self-Reported Mental Health Problems Among Adults Born Preterm: A Meta-Analysis. Pediatrics.

[76] Porges S.W., Furman S.A. (2011). The Early Development of the Autonomic Nervous System Provides a Neural Platform for Social Behavior: A Polyvagal Perspective. Infant Child Dev..

[77] Fyfe K.L., Yiallourou S.R., Wong F.Y., Odoi A., Walker A.M., Horne R.S.C. (2015). The Effect of Gestational Age at Birth on Post-Term Maturation of Heart Rate Variability. Sleep.

[78] Yiallourou S.R., Witcombe N.B., Sands S.A., Walker A.M., Horne R.S.C. (2013). The Development of Autonomic Cardiovascular Control Is Altered by Preterm Birth. Early Hum. Dev..

[79] Wilkowska A., Rynkiewicz A., Wdowczyk J., Landowski J., Cubała W.J. (2019). Heart Rate Variability and Incidence of Depression during the First Six Months Following First Myocardial Infarction. Neuropsychiatr. Dis. Treat..

[80] Yu L.-C., Lin I.-M., Fan S.-Y., Chien C.-L., Lin T.-H. (2018). One-Year Cardiovascular Prognosis of the Randomized, Controlled, Short-Term Heart Rate Variability Biofeedback Among Patients with Coronary Artery Disease. Int. J. Behav. Med..

[81] Limmer A., Laser M., Schütz A. (2022). Mobile Heart Rate Variability Biofeedback as a Complementary Intervention After Myocardial Infarction: A Randomized Controlled Study. Int. J. Behav. Med..

[82] Xu X., Qiao J., Wang Y., Liu Z., Liu Z., Tan W., Wang C., Peng C., Cheng S., Han X. (2025). PENG-Based Self-Powered Transcutaneous Auricular Vagus Nerve Stimulation Attenuated Myocardial Infarction-Induced Heart-Brain Remodeling via Ameliorating the Neuroinflammatory Response in Central Amygdala. Int. Immunopharmacol..

[83] Ciccarelli M., Sorriento D., Fiordelisi A., Gambardella J., Franco A., Del Giudice C., Sala M., Monti M.G., Bertamino A., Campiglia P. (2020). Pharmacological Inhibition of GRK2 Improves Cardiac Metabolism and Function in Experimental Heart Failure. ESC Heart Fail..

[84] Machhada A., Hosford P.S., Dyson A., Ackland G.L., Mastitskaya S., Gourine A.V. (2020). Optogenetic Stimulation of Vagal Efferent Activity Preserves Left Ventricular Function in Experimental Heart Failure. JACC Basic Transl. Sci..

[85] Machhada A., Trapp S., Marina N., Stephens R.C.M., Whittle J., Lythgoe M.F., Kasparov S., Ackland G.L., Gourine A.V. (2017). Vagal Determinants of Exercise Capacity. Nat. Commun..

[86] Tian X., Wang Q., Guo R., Xu L., Chen Q.M., Hou Y. (2016). Effects of Paroxetine-Mediated Inhibition of GRK2 Expression on Depression and Cardiovascular Function in Patients with Myocardial Infarction. Neuropsychiatr. Dis. Treat..

[87] Ngampramuan S., Tungtong P., Mukda S., Jariyavilas A., Sakulisariyaporn C. (2018). Evaluation of Autonomic Nervous System, Saliva Cortisol Levels, and Cognitive Function in Major Depressive Disorder Patients. Depress. Res. Treat..

[88] Morais-Silva G., Costa-Ferreira W., Gomes-de-Souza L., Pavan J.C., Crestani C.C., Marin M.T. (2019). Cardiovascular Outcomes Related to Social Defeat Stress: New Insights from Resilient and Susceptible Rats. Neurobiol. Stress..

[89] Euteneuer F., Neuert M., Salzmann S., Fischer S., Ehlert U., Rief W. (2023). Does Psychological Treatment of Major Depression Reduce Cardiac Risk Biomarkers? An Exploratory Randomized Controlled Trial. Psychol. Med..

[90] Helman T.J., Headrick J.P., Peart J.N., Stapelberg N.J.C. (2022). Central and Cardiac Stress Resilience Consistently Linked to Integrated Immuno-Neuroendocrine Responses across Stress Models in Male Mice. Eur. J. Neurosci..

[91] Liu X., Qu C., Yang H., Shi S., Zhang C., Zhang Y., Liang J., Yang B. (2018). Chronic Stimulation of the Sigma-1 Receptor Ameliorates Autonomic Nerve Dysfunction and Atrial Fibrillation Susceptibility in a Rat Model of Depression. Am. J. Physiol. Heart Circ. Physiol..

[92] Hu M.X., Milaneschi Y., Lamers F., Nolte I.M., Snieder H., Dolan C.V., Penninx B.W.J.H., de Geus E.J.C. (2019). The Association of Depression and Anxiety with Cardiac Autonomic Activity: The Role of Confounding Effects of Antidepressants. Depress. Anxiety.

[93] Devarajan A., Wang K., Lokhandwala Z.A., Emamimeybodi M., Shannon K., Tompkins J.D., Hevener A.L., Lusis A.J., Abel E.D., Vaseghi M. (2024). Myocardial Infarction Causes Sex-Dependent Dysfunction in Vagal Sensory Glutamatergic Neurotransmission That Is Mitigated by 17β-Estradiol. JCI Insight.

[94] Zhang K., Pan X., Wang F., Ma J., Su G., Dong Y., Yang J., Wu C. (2016). Baicalin Promotes Hippocampal Neurogenesis via SGK1- and FKBP5-Mediated Glucocorticoid Receptor Phosphorylation in a Neuroendocrine Mouse Model of Anxiety/Depression. Sci. Rep..

[95] Deng F., Li X., Tang C., Chen J., Fan B., Liang J., Zhen X., Tao R., Zhang S., Cong Z. (2022). Mechanisms of Xiong-Pi-Fang in Treating Coronary Heart Disease Associated with Depression: A Systematic Pharmacology Strategy and in Vivo Pharmacological Validation. J. Ethnopharmacol..

[96] Verma H., Bhattacharjee A., Shivavedi N., Nayak P.K. (2022). Evaluation of Rosmarinic Acid against Myocardial Infarction in Maternally Separated Rats. Naunyn Schmiedeberg’s Arch. Pharmacol..

[97] Park J.-Y., Kim J.-W., Kang H.-J., Choi W., Lee J.-Y., Kim S.-W., Shin I.-S., Ahn Y., Jeong M.H., Kim J.-M. (2023). Effect Modification of Cortisol on the Associations Between Obsessive-Compulsive Symptoms on Suicidality in Patients With Acute Coronary Syndrome. Psychiatry Investig..

[98] Wilkowska A., Rynkiewicz A., Wdowczyk J., Landowski J. (2019). Morning and Afternoon Serum Cortisol Level in Patients with Post-Myocardial Infarction Depression. Cardiol. J..

[99] Poole L., Kidd T., Ronaldson A., Leigh E., Jahangiri M., Steptoe A. (2016). Depression 12-Months after Coronary Artery Bypass Graft Is Predicted by Cortisol Slope over the Day. Psychoneuroendocrinology.

[100] Bruns B., Daub R., Schmitz T., Hamze-Sinno M., Spaich S., Dewenter M., Schwale C., Gass P., Vogt M., Katus H. (2022). Forebrain Corticosteroid Receptors Promote Post-Myocardial Infarction Depression and Mortality. Basic Res. Cardiol..

[101] Kun W., Jie Z., Shuai C., Xin W.U., Guoqi Z., Shengbing W.U., Meiqi Z. (2023). Electroacupuncture Ameliorates Cardiac Dysfunction in Myocardial Ischemia Model Rats: A Potential Role of the Hypothalamic-Pituitary-Adrenal Axis. J. Tradit. Chin. Med..

[102] Ma Y., Yang X., Villalba N., Chatterjee V., Reynolds A., Spence S., Wu M.H., Yuan S.Y. (2022). Circulating Lymphocyte Trafficking to the Bone Marrow Contributes to Lymphopenia in Myocardial Infarction. Am. J. Physiol. Heart Circ. Physiol..

[103] Kang H.-J., Stewart R., Kim J.-W., Kim S.-W., Shin I.-S., Kim M.-C., Hong Y.J., Ahn Y., Shin M.-G., Jeong M.H. (2020). Synergistic Effects of Depression and NR3C1 Methylation on Prognosis of Acute Coronary Syndrome. Sci. Rep..

[104] Nikkheslat N., McLaughlin A.P., Hastings C., Zajkowska Z., Nettis M.A., Mariani N., Enache D., Lombardo G., Pointon L., Cowen P.J. (2020). Childhood Trauma, HPA Axis Activity and Antidepressant Response in Patients with Depression. Brain Behav. Immun..

[105] Gan L., Li N., Heizati M., Lin M., Zhu Q., Hong J., Wu T., Tong L., Xiamili Z., Lin Y. (2022). Diurnal Cortisol Features with Cardiovascular Disease in Hypertensive Patients: A Cohort Study. Eur. J. Endocrinol..

[106] Labad J., Soria V., Salvat-Pujol N., Segalàs C., Real E., Urretavizcaya M., de Arriba-Arnau A., Ferrer A., Crespo J.M., Jiménez-Murcia S. (2018). Hypothalamic-Pituitary-Adrenal Axis Activity in the Comorbidity between Obsessive-Compulsive Disorder and Major Depression. Psychoneuroendocrinology.

[107] Nowacki J., Wingenfeld K., Kaczmarczyk M., Chae W.R., Salchow P., Abu-Tir I., Piber D., Hellmann-Regen J., Otte C. (2020). Steroid Hormone Secretion after Stimulation of Mineralocorticoid and NMDA Receptors and Cardiovascular Risk in Patients with Depression. Transl. Psychiatry.

[108] Haj-Mirzaian A., Ramezanzadeh K., Shariatzadeh S., Tajik M., Khalafi F., Tafazolimoghadam A., Radmard M., Rahbar A., Pirri F., Kazemi K. (2021). Role of Hypothalamic-Pituitary Adrenal-Axis, Toll-like Receptors, and Macrophage Polarization in Pre-Atherosclerotic Changes Induced by Social Isolation Stress in Mice. Sci. Rep..

[109] Scarborough J., Mueller F.S., Weber-Stadlbauer U., Mattei D., Opitz L., Cattaneo A., Richetto J. (2021). A Novel Murine Model to Study the Impact of Maternal Depression and Antidepressant Treatment on Biobehavioral Functions in the Offspring. Mol. Psychiatry.

[110] Holsboer F. (2000). The Corticosteroid Receptor Hypothesis of Depression. Neuropsychopharmacology.

[111] Bunevicius A., Gintauskiene V., Podlipskyte A., Zaliunas R., Brozaitiene J., Prange A.J., Bunevicius R. (2012). Fatigue in Patients with Coronary Artery Disease: Association with Thyroid Axis Hormones and Cortisol. Psychosom. Med..

[112] Nikkheslat N., Zunszain P.A., Horowitz M.A., Barbosa I.G., Parker J.A., Myint A.-M., Schwarz M.J., Tylee A.T., Carvalho L.A., Pariante C.M. (2015). Insufficient Glucocorticoid Signaling and Elevated Inflammation in Coronary Heart Disease Patients with Comorbid Depression. Brain Behav. Immun..

[113] Malan L., Schutte C.E., Alkerwi A., Stranges S., Malan N.T. (2017). Hypothalamic-Pituitary-Adrenal-Axis Dysregulation and Double Product Increases Potentiate Ischemic Heart Disease Risk in a Black Male Cohort: The SABPA Study. Hypertens. Res..

[114] von Känel R., Schmid J.-P., Abbas C.C., Gander M.-L., Saner H., Begré S. (2010). Stress Hormones in Patients with Posttraumatic Stress Disorder Caused by Myocardial Infarction and Role of Comorbid Depression. J. Affect. Disord..

[115] Messerli-Bürgy N., Molloy G.J., Wikman A., Perkins-Porras L., Randall G., Steptoe A. (2012). Cortisol Levels and History of Depression in Acute Coronary Syndrome Patients. Psychol. Med..

[116] Kwok M.K., Kawachi I., Rehkopf D., Schooling C.M. (2020). The Role of Cortisol in Ischemic Heart Disease, Ischemic Stroke, Type 2 Diabetes, and Cardiovascular Disease Risk Factors: A Bi-Directional Mendelian Randomization Study. BMC Med..

[117] Fang L., Moore X.-L., Dart A.M., Wang L.-M. (2015). Systemic Inflammatory Response Following Acute Myocardial Infarction. J. Geriatr. Cardiol..

[118] Frangogiannis N.G. (2014). The Inflammatory Response in Myocardial Injury, Repair, and Remodelling. Nat. Rev. Cardiol..

[119] Miller A.H., Raison C.L. (2016). The Role of Inflammation in Depression: From Evolutionary Imperative to Modern Treatment Target. Nat. Rev. Immunol..

[120] Galea I. (2021). The Blood-Brain Barrier in Systemic Infection and Inflammation. Cell. Mol. Immunol..

[121] Huppert J., Closhen D., Croxford A., White R., Kulig P., Pietrowski E., Bechmann I., Becher B., Luhmann H.J., Waisman A. (2010). Cellular Mechanisms of IL-17-Induced Blood-Brain Barrier Disruption. FASEB J..

[122] Traub J., Grondey K., Gassenmaier T., Schmitt D., Fette G., Frantz S., Boivin-Jahns V., Jahns R., Störk S., Stoll G. (2022). Sustained Increase in Serum Glial Fibrillary Acidic Protein after First ST-Elevation Myocardial Infarction. Int. J. Mol. Sci..

[123] Baysak E., Yildirim C., Sayar N., Sayar M.K., Halaris A., Aricioglu F. (2023). The Possible Role of NLRP3 Inflammasome in Depression and Myocardial Infarction Comorbidity. J. Pers. Med..

[124] Lu H., Yang Q., Zhang Y. (2022). The Relation of Common Inflammatory Cytokines with Anxiety and Depression and Their Values in Estimating Cardiovascular Outcomes in Coronary Heart Disease Patients. J. Clin. Lab. Anal..

[125] Moludi J., Alizadeh M., Mohammadzad M.H.S., Davari M. (2019). The Effect of Probiotic Supplementation on Depressive Symptoms and Quality of Life in Patients After Myocardial Infarction: Results of a Preliminary Double-Blind Clinical Trial. Psychosom. Med..

[126] Li C., Wan S., Li W., Wang Y., Li B., Chen Y., Sun P., Lyu J. (2022). Higher Neutrophil to Lymphocyte Ratio at Admission Is Association with Post-PCI Depressive Symptoms in Patients with ACS. Neuropsychiatr. Dis. Treat..

[127] Najjar F., Ahmad M., Lagace D., Leenen F.H.H. (2019). Role of Myocardial Infarction-Induced Neuroinflammation for Depression-Like Behavior and Heart Failure in Ovariectomized Female Rats. Neuroscience.

[128] Wang H.-W., Ahmad M., Jadayel R., Najjar F., Lagace D., Leenen F.H.H. (2019). Inhibition of Inflammation by Minocycline Improves Heart Failure and Depression-like Behaviour in Rats after Myocardial Infarction. PLoS ONE.

[129] Yang Y., Huang T., Zhang H., Li X., Shi S., Tian X., Huang Z., Zhang R., Liu Z., Cheng Y. (2023). Formononetin Improves Cardiac Function and Depressive Behaviours in Myocardial Infarction with Depression by Targeting GSK-3β to Regulate Macrophage/Microglial Polarization. Phytomedicine.

[130] Wang Y., Chen Y., Li B., Zhou Y., Guan J., Huang F., Wu J., Dong Y., Sun P., Tian X. (2024). The Antidepressant Effect of Shexiang Baoxin Pills on Myocardial Infarction Rats with Depression May Be Achieved through the Inhibition of the NLRP3 Inflammasome Pathway. Brain Behav..

[131] Sun Y., Wang Z., Hou J., Shi J., Tang Z., Wang C., Zhao H. (2022). Shuangxinfang Prevents S100A9-Induced Macrophage/Microglial Inflammation to Improve Cardiac Function and Depression-Like Behavior in Rats After Acute Myocardial Infarction. Front. Pharmacol..

[132] Sun Y., Wang Z., Wang C., Tang Z., Zhao H. (2021). Psycho-Cardiology Therapeutic Effects of Shuangxinfang in Rats with Depression-Behavior Post Acute Myocardial Infarction: Focus on Protein S100A9 from Proteomics. Biomed. Pharmacother..

[133] Ge Y., Xu W., Zhang L., Liu M. (2020). Ginkgolide B Attenuates Myocardial Infarction-Induced Depression-like Behaviors via Repressing IL-1β in Central Nervous System. Int. Immunopharmacol..

[134] Su J., Wang J., Ma Y., Li Q., Yang Y., Huang L., Wang H., Li H., Wang Z., Tong J. (2019). Inflammation Associated with Chronic Heart Failure Leads to Enhanced Susceptibility to Depression. FEBS J..

[135] Tang X., Liu R., Zhang Y., Zhu L., Shi W., Shan Y., Wu S., Li Y., Liu G., Ma W. (2022). Downregulation of Interleukin-1 Beta via Jmjd3 Inhibition Improves Post-Myocardial Infarction Depression. Cardiovasc. Diagn. Ther..

[136] Zhang Y., Wang X., Li Y., Liu R., Pan J., Tang X., Sun S., Liu J., Ma W. (2021). Human Umbilical Cord Mesenchymal Stem Cells Ameliorate Depression by Regulating Jmjd3 and Microglia Polarization in Myocardial Infarction Mice. Psychopharmacology.

[137] Sorci G., Bianchi R., Riuzzi F., Tubaro C., Arcuri C., Giambanco I., Donato R. (2010). S100B Protein, A Damage-Associated Molecular Pattern Protein in the Brain and Heart, and Beyond. Cardiovasc. Psychiatry Neurol..

[138] Abel W.M., Scanlan L.N., Horne C.E., Crane P.B. (2022). Factors Associated with Myocardial Infarction Reoccurrence. J. Cardiovasc. Nurs..

[139] Mester A., Benedek T., Ratiu M., Morariu M., Benedek A., Hodas R., Opincariu D., Rat N., Chitu M., Benedek I. (2019). 490 Persistently Increased Inflammatory Status in the First Days Post MI Is Associated with Larger Myocardial Scar, Higher Transmurality Extent and Deterioration of Ventricular Function at 1 Month. Eur. Heart J. Cardiovasc. Imaging.

[140] Petyunina O.V., Kopytsya M.P., Vyshnevska I.R. (2021). Interactions between Macrophage Inhibitor Factor and Emotional Distress in Patients with St-Segment Elevation Myocardial Infarction. Atherosclerosis.

[141] Qian L., Zhang S., Lin C., Lin J., Li Z. (2024). Depression Exacerbates Myocardial Ischemia-Reperfusion Injury in Mice via CNR2 Gene and MIF-AMPK Signaling Pathway. Int. J. Cardiol..

[142] von Känel R., Rosselet K., Gessler K., Haeussler A., Aschmann J., Rodriguez H., Dzemali O. (2022). Preoperative Depression and Anxiety as Predictors of Postoperative C-Reactive Protein Levels in Patients Undergoing Cardiac Surgery: A Prospective Observational Study. Swiss Med. Wkly..

[143] Min J.J., Nam K., Kim T.K., Kim H.J., Seo J.H., Hwang H.Y., Kim K.B., Murkin J.M., Hong D.M., Jeon Y. (2014). Relationship between Early Postoperative C-Reactive Protein Elevation and Long-Term Postoperative Major Adverse Cardiovascular and Cerebral Events in Patients Undergoing off-Pump Coronary Artery Bypass Graft Surgery: A Retrospective Study. Br. J. Anaesth..

[144] Hariri A.R., Mattay V.S., Tessitore A., Kolachana B., Fera F., Goldman D., Egan M.F., Weinberger D.R. (2002). Serotonin Transporter Genetic Variation and the Response of the Human Amygdala. Science.

[145] Lesch K.P., Bengel D., Heils A., Sabol S.Z., Greenberg B.D., Petri S., Benjamin J., Müller C.R., Hamer D.H., Murphy D.L. (1996). Association of Anxiety-Related Traits with a Polymorphism in the Serotonin Transporter Gene Regulatory Region. Science.

[146] Caspi A., Sugden K., Moffitt T.E., Taylor A., Craig I.W., Harrington H., McClay J., Mill J., Martin J., Braithwaite A. (2003). Influence of Life Stress on Depression: Moderation by a Polymorphism in the 5-HTT Gene. Science.

[147] Popp S., Schmitt-Böhrer A., Langer S., Hofmann U., Hommers L., Schuh K., Frantz S., Lesch K.-P., Frey A. (2021). 5-HTT Deficiency in Male Mice Affects Healing and Behavior after Myocardial Infarction. J. Clin. Med..

[148] Luong H., Singh S., Patil M., Krishnamurthy P. (2021). Cardiac Glycosaminoglycans and Structural Alterations during Chronic Stress-Induced Depression-like Behavior in Mice. Am. J. Physiol. Heart Circ. Physiol..

[149] Lockhart M., Wirrig E., Phelps A., Wessels A. (2011). Extracellular Matrix and Heart Development. Birth Defects Res. A Clin. Mol. Teratol..

[150] O’Callaghan P., Zhang X., Li J.-P. (2018). Heparan Sulfate Proteoglycans as Relays of Neuroinflammation. J. Histochem. Cytochem..

[151] Baranyi A., Enko D., Meinitzer A., Von Lewinski D., Rothenhäusler H.-B., Harpf L., Traninger H., Obermayer-Pietsch B., Harb B.M., Schweinzer M. (2022). Myeloperoxidase as a Potential Biomarker of Acute-Myocardial-Infarction-Induced Depression and Suppression of the Innate Immune System. Antioxidants.

[152] Zhang L., Li G., Tao S., Xia P., Chaudhry N., Kaura S., Stone S.S., Liu M. (2022). Ginkgo Biloba Extract Reduces Cardiac and Brain Inflammation in Rats Fed a HFD and Exposed to Chronic Mental Stress through NF-κB Inhibition. Mediat. Inflamm..

[153] Yu C., Zhang F., Zhang L., Li J., Tang S., Li X., Peng M., Zhao Q., Zhu X. (2023). A Bioinformatics Approach to Identifying the Biomarkers and Pathogenesis of Major Depressive Disorder Combined with Acute Myocardial Infarction. Am. J. Transl. Res..

[154] Wang M., Cheng L., Gao Z., Li J., Ding Y., Shi R., Xiang Q., Chen X. (2023). Investigation of the Shared Molecular Mechanisms and Hub Genes between Myocardial Infarction and Depression. Front. Cardiovasc. Med..

[155] Nurillaeva N.M., Abdumalikova F.B. (2021). Predictive Importance of Psycho-Emotional Syndrome of Patients with Coronary Heart Disease in the Violation of Platelet Hemostatic System. Atherosclerosis.

[156] von Känel R., Pazhenkottil A.P., Meister-Langraf R.E., Znoj H., Schmid J.-P., Zuccarella-Hackl C., Barth J., Schnyder U., Princip M. (2021). Longitudinal Association between Cognitive Depressive Symptoms and D-Dimer Levels in Patients Following Acute Myocardial Infarction. Clin. Cardiol..

[157] Deter H.-C., Orth-Gomér K., Rauch-Kröhnert U., Albus C., Ladwig K.-H., Söllner W., de Zwaan M., Grün A.-S., Ronel J., Hellmich M. (2021). Depression, Anxiety, and Vital Exhaustion Are Associated with pro-Coagulant Markers in Depressed Patients with Coronary Artery Disease—A Cross Sectional and Prospective Secondary Analysis of the SPIRR-CAD Trial. J. Psychosom. Res..

[158] Rieder M., Gauchel N., Bode C., Duerschmied D. (2021). Serotonin: A Platelet Hormone Modulating Cardiovascular Disease. J. Thromb. Thrombolysis.

[159] Hoirisch-Clapauch S., Nardi A.E., Gris J.-C., Brenner B. (2014). Are the Antiplatelet and Profibrinolytic Properties of Selective Serotonin-Reuptake Inhibitors Relevant to Their Brain Effects?. Thromb. Res..

[160] Serebruany V.L., Gurbel P.A., O’Connor C.M. (2001). Platelet Inhibition by Sertraline and N-Desmethylsertraline: A Possible Missing Link between Depression, Coronary Events, and Mortality Benefits of Selective Serotonin Reuptake Inhibitors. Pharmacol. Res..

[161] Youssef M.M., Underwood M.D., Huang Y.-Y., Hsiung S.-C., Liu Y., Simpson N.R., Bakalian M.J., Rosoklija G.B., Dwork A.J., Arango V. (2018). Association of BDNF Val66Met Polymorphism and Brain BDNF Levels with Major Depression and Suicide. Int. J. Neuropsychopharmacol..

[162] Amadio P., Colombo G.I., Tarantino E., Gianellini S., Ieraci A., Brioschi M., Banfi C., Werba J.P., Parolari A., Lee F.S. (2017). BDNFVal66met Polymorphism: A Potential Bridge between Depression and Thrombosis. Eur. Heart J..

[163] Petyunina O.V., Kopytsya M.P., Berezin A.E. (2020). Brain-Derived Neurotrophic Factor Gene Polymorphism in Post-ST-Elevation Myocardial Infarction Patients Undergoing Primary Percutaneous Intervention. Biomed. Res. Ther..

[164] Sandrini L., Castiglioni L., Amadio P., Werba J.P., Eligini S., Fiorelli S., Zarà M., Castiglioni S., Bellosta S., Lee F.S. (2020). Impact of BDNF Val66Met Polymorphism on Myocardial Infarction: Exploring the Macrophage Phenotype. Cells.

[165] Sandrini L., Amadio P., Ieraci A., Malara A., Werba J.P., Soprano P.M., Balduini A., Zarà M., Bonomi A., Veglia F. (2022). The A2-Adrenergic Receptor Pathway Modulating Depression Influences the Risk of Arterial Thrombosis Associated with BDNFVal66Met Polymorphism. Biomed. Pharmacother..

[166] Verhagen M., van der Meij A., van Deurzen P.A.M., Janzing J.G.E., Arias-Vásquez A., Buitelaar J.K., Franke B. (2010). Meta-Analysis of the BDNF Val66Met Polymorphism in Major Depressive Disorder: Effects of Gender and Ethnicity. Mol. Psychiatry.

[167] Wang Y., Li O., Li N., Sha Z., Zhao Z., Xu J. (2023). Association between the BDNF Val66Met Polymorphism and Major Depressive Disorder: A Systematic Review and Meta-Analysis. Front. Psychiatry.

[168] Geiser F., Urbach A.S., Harbrecht U., Conrad R., Pötzsch B., Amann N., Kiesewetter K., Sieke A., Wolffs K., Skowasch D. (2017). Anxiety and Depression in Patients Three Months after Myocardial Infarction: Association with Markers of Coagulation and the Relevance of Age. J. Psychosom. Res..

[169] Empana J.P., Sykes D.H., Luc G., Juhan-Vague I., Arveiler D., Ferrieres J., Amouyel P., Bingham A., Montaye M., Ruidavets J.B. (2005). Contributions of Depressive Mood and Circulating Inflammatory Markers to Coronary Heart Disease in Healthy European Men: The Prospective Epidemiological Study of Myocardial Infarction (PRIME). Circulation.

[170] Piantella S., Dragano N., Marques M., McDonald S.J., Wright B.J. (2021). Prospective Increases in Depression Symptoms and Markers of Inflammation Increase Coronary Heart Disease Risk—The Whitehall II Cohort Study. J. Psychosom. Res..

[171] Sama J., Vaidya D., Mukherjee M., Williams M. (2021). Effects of Clinical Depression on Left Ventricular Dysfunction in Patients with Acute Coronary Syndrome. J. Thromb. Thrombolysis.

[172] Shimokhina N.Y., Savchenko A.A., Petrova M.M. (2020). Peculiarities of Platelet Metabolism in Patients with Acute Coronary Syndrome with Anxiety-Depressive Disorders and Informativity of Enzymes in the Forecast of Development of Cardiovascular Complications. Pharmaceuticals.

[173] Jiang B., Wu R.-M., Li H.-D., Li K., Li H., Dang W.-Z., Feng G.-Z., Bao W.-L., Ye G., Shen X.-Y. (2022). Yixin Ningshen Tablet Alleviates Comorbidity of Myocardial Infarction and Depression by Enhancing Myocardial Energy Metabolism and Increasing Availability of Monoamine Neurotransmitter. Chin. J. Integr. Med..

[174] Hamdan D.I., Hafez S.S., Hassan W.H.B., Morsi M.M., Khalil H.M.A., Ahmed Y.H., Ahmed-Farid O.A., El-Shiekh R.A. (2022). Chemical Profiles with Cardioprotective and Anti-Depressive Effects of Morus Macroura Miq. Leaves and Stem Branches Dichloromethane Fractions on Isoprenaline Induced Post-MI Depression. RSC Adv..

[175] Liu M.-Y., Ren Y.-P., Zhang L.-J., Ding J.Y. (2016). Pretreatment with Ginseng Fruit Saponins Affects Serotonin Expression in an Experimental Comorbidity Model of Myocardial Infarction and Depression. Aging Dis..

[176] He D.-F., Ren Y.-P., Liu M.-Y. (2016). Effects of Ginseng Fruit Saponins on Serotonin System in Sprague-Dawley Rats with Myocardial Infarction, Depression, and Myocardial Infarction Complicated with Depression. Chin. Med. J..

[177] Manjarrez-Gutiérrez G., Ramírez-Campillo R., Borrayo-Sánchez G., Hernández-Rodríguez J. (2013). Disturbance of Serotonergic Neurotransmission in Patients with Postmyocardial Infarction and Depression. Metab. Brain Dis..

[178] Shaw D.M., Macsweeney D.A., Hewland R., Johnson A.L. (1975). Tricyclic Antidepressants and Tryptophan in Unipolar Depression. Psychol. Med..

[179] Fraer M., Kilic F. (2015). Serotonin: A Different Player in Hypertension-Associated Thrombosis. Hypertension.

[180] Brattelid T., Qvigstad E., Moltzau L.R., Bekkevold S.V.S., Sandnes D.L., Birkeland J.A.K., Skomedal T., Osnes J.-B., Sjaastad I., Levy F.O. (2012). The Cardiac Ventricular 5-HT4 Receptor Is Functional in Late Foetal Development and Is Reactivated in Heart Failure. PLoS ONE.

[181] Kim Y., Lee Y.S., Kim M.G., Song Y.-K., Kim Y., Jang H., Kim J.H., Han N., Ji E., Kim I.-W. (2019). The Effect of Selective Serotonin Reuptake Inhibitors on Major Adverse Cardiovascular Events: A Meta-Analysis of Randomized-Controlled Studies in Depression. Int. Clin. Psychopharmacol..

[182] Rami M., Guillamat-Prats R., Rinne P., Salvermoser M., Ring L., Bianchini M., Blanchet X., Megens R.T.A., Döring Y., Walzog B. (2018). Chronic Intake of the Selective Serotonin Reuptake Inhibitor Fluoxetine Enhances Atherosclerosis. Arter. Thromb. Vasc. Biol..

[183] Liu M.-Y., Ren Y.-P., Wei W.-L., Tian G.-X., Li G. (2015). Changes of Serotonin (5-HT), 5-HT2A Receptor, and 5-HT Transporter in the Sprague-Dawley Rats of Depression, Myocardial Infarction and Myocardial Infarction Co-Exist with Depression. Chin. Med. J..

[184] Li C., Cai Q., Su Z., Chen Z., Cao J., Xu F. (2023). Could Peripheral 5-HT Level Be Used as a Biomarker for Depression Diagnosis and Treatment? A Narrative Minireview. Front. Pharmacol..

[185] Mauler M., Herr N., Schoenichen C., Witsch T., Marchini T., Härdtner C., Koentges C., Kienle K., Ollivier V., Schell M. (2019). Platelet Serotonin Aggravates Myocardial Ischemia/Reperfusion Injury via Neutrophil Degranulation. Circulation.

[186] Zhang L., Liu M., Chen H., Li Y., Rao P. (2025). Antidepressant in Treating Myocardial Infarction Complicated with Depression via 5-HT/Inflammation from Heart to Brain. J. Affect. Disord..

[187] Zhang L.-J., Zeng X.-T., Zhao M.-J., He D.-F., Liu J.-Y., Liu M.-Y. (2020). The Important Effect of 5-HTTLPR Polymorphism on the Risk of Depression in Patients with Coronary Heart Disease: A Meta-Analysis. BMC Cardiovasc. Disord..

[188] Doggrell S.A. (2003). The Role of 5-HT on the Cardiovascular and Renal Systems and the Clinical Potential of 5-HT Modulation. Expert. Opin. Investig. Drugs.

[189] Fernandes N., Prada L., Rosa M.M., Ferreira J.J., Costa J., Pinto F.J., Caldeira D. (2021). The Impact of SSRIs on Mortality and Cardiovascular Events in Patients with Coronary Artery Disease and Depression: Systematic Review and Meta-Analysis. Clin. Res. Cardiol..

[190] Karlsen H.R., Løchen M.-L., Langvik E. (2023). Antidepressant Use and Risk of Myocardial Infarction: A Longitudinal Investigation of Sex-Specific Associations in the HUNT Study. Psychosom. Med..

[191] Li M., Kwok M.K., Fong S.S.M., Schooling C.M. (2020). Effects of Tryptophan, Serotonin, and Kynurenine on Ischemic Heart Diseases and Its Risk Factors: A Mendelian Randomization Study. Eur. J. Clin. Nutr..

[192] Bahr F.S., Ricke-Hoch M., Ponimaskin E., Müller F.E. (2024). Serotonin Receptors in Myocardial Infarction: Friend or Foe?. ACS Chem. Neurosci..

[193] Han W., Wei Z., Dang R., Guo Y., Zhang H., Geng C., Wang C., Feng Q., Jiang P. (2020). Angiotensin-Ⅱ and Angiotensin-(1–7) Imbalance Affects Comorbidity of Depression and Coronary Heart Disease. Peptides.

[194] Mathews R., Wang T.Y., Honeycutt E., Henry T.D., Zettler M., Chang M., Fonarow G.C., Peterson E.D., TRANSLATE-ACS Study Investigators (2015). Persistence with Secondary Prevention Medications after Acute Myocardial Infarction: Insights from the TRANSLATE-ACS Study. Am. Heart J..

[195] Lima B.B., Hammadah M., Kim J.H., Uphoff I., Shah A., Levantsevych O., Almuwaqqat Z., Moazzami K., Sullivan S., Ward L. (2019). Association of Transient Endothelial Dysfunction Induced by Mental Stress With Major Adverse Cardiovascular Events in Men and Women with Coronary Artery Disease. JAMA Cardiol..

[196] Di Stefano R., Felice F., Pini S., Mazzotta G., Bovenzi F.M., Bertoli D., Abelli M., Borelli L., Cardini A., Lari L. (2014). Impact of Depression on Circulating Endothelial Progenitor Cells in Patients with Acute Coronary Syndromes: A Pilot Study. J. Cardiovasc. Med..

[197] Madva E.N., Celano C.M., Smith D.M., Januzzi J.L., Huffman J.C. (2021). Recurrent versus New-Onset Depressive Symptoms: Relationships with Biomarkers of Cardiovascular Health Following Acute Coronary Syndrome. J. Psychosom. Res..

[198] Yammine L., Frazier L., Padhye N.S., Sanner J.E., Burg M.M. (2017). Two-Year Prognosis after Acute Coronary Syndrome in Younger Patients: Association with Feeling Depressed in the Prior Year, and BDI-II Score and Endothelin-1. J. Psychosom. Res..

[199] Yammine L., Frazier L., Padhye N.S., Burg M.M., Meininger J.C. (2014). Severe Depressive Symptoms Are Associated with Elevated Endothelin-1 in Younger Patients with Acute Coronary Syndrome. J. Psychosom. Res..

[200] Jazdzewski K., Murray E.L., Franssila K., Jarzab B., Schoenberg D.R., de la Chapelle A. (2008). Common SNP in Pre-miR-146a Decreases Mature miR Expression and Predisposes to Papillary Thyroid Carcinoma. Proc. Natl. Acad. Sci. USA.

[201] Zhang X., Huo Q., Sun W., Zhang C., Wu Z., Xing B., Li Q. (2018). Rs2910164 in microRNA-146a Confers an Elevated Risk of Depression in Patients with Coronary Artery Disease by Modulating the Expression of NOS1. Mol. Med. Rep..

[202] Gao S.-F., Lu Y.-R., Shi L.-G., Wu X.-Y., Sun B., Fu X.-Y., Luo J.-H., Bao A.-M. (2014). Nitric Oxide Synthase and Nitric Oxide Alterations in Chronically Stressed Rats: A Model for Nitric Oxide in Major Depressive Disorder. Psychoneuroendocrinology.

[203] Steinert J.R., Chernova T., Forsythe I.D. (2010). Nitric Oxide Signaling in Brain Function, Dysfunction, and Dementia. Neuroscientist.

[204] Zhou Q.-G., Zhu L.-J., Chen C., Wu H.-Y., Luo C.-X., Chang L., Zhu D.-Y. (2011). Hippocampal Neuronal Nitric Oxide Synthase Mediates the Stress-Related Depressive Behaviors of Glucocorticoids by Downregulating Glucocorticoid Receptor. J. Neurosci..

[205] Pögün S., Kuhar M.J. (1994). Regulation of Neurotransmitter Reuptake by Nitric Oxide. Ann. N. Y. Acad. Sci..

[206] Chen H., Zhang L., Li Y., Meng X., Chi Y., Liu M. (2024). Gut Microbiota and Its Metabolites: The Emerging Bridge Between Coronary Artery Disease and Anxiety and Depression?. Aging Dis..

[207] Wang Q., Wang X., Lv Y., Yang C., Zhou C., Wang L. (2021). Changes in Rats’ Gut Microbiota Composition Caused by Induced Chronic Myocardial Infarction Lead to Depression-Like Behavior. Front. Microbiol..

[208] Liao L., Zhang L., Yang C., Wang T., Feng L., Peng C., Long Y., Dai G., Chang L., Wei Y. (2024). Sotagliflozin Attenuates Cardiac Dysfunction and Depression-like Behaviors in Mice with Myocardial Infarction through the Gut-Heart-Brain Axis. Neurobiol. Dis..

[209] Sun B., Ma T., Li Y., Yang N., Li B., Zhou X., Guo S., Zhang S., Kwok L.-Y., Sun Z. (2022). Bifidobacterium Lactis Probio-M8 Adjuvant Treatment Confers Added Benefits to Patients with Coronary Artery Disease via Target Modulation of the Gut-Heart/-Brain Axes. mSystems.

[210] Moludi J., Khedmatgozar H., Nachvak S.M., Abdollahzad H., Moradinazar M., Sadeghpour Tabaei A. (2022). The Effects of Co-Administration of Probiotics and Prebiotics on Chronic Inflammation, and Depression Symptoms in Patients with Coronary Artery Diseases: A Randomized Clinical Trial. Nutr. Neurosci..

[211] Gagnon E., Mitchell P.L., Manikpurage H.D., Abner E., Taba N., Esko T., Ghodsian N., Thériault S., Mathieu P., Arsenault B.J. (2023). Impact of the Gut Microbiota and Associated Metabolites on Cardiometabolic Traits, Chronic Diseases and Human Longevity: A Mendelian Randomization Study. J. Transl. Med..

[212] Verma H., Shivavedi N., Tej G.N.V.C., Kumar M., Nayak P.K. (2022). Prophylactic Administration of Rosmarinic Acid Ameliorates Depression-Associated Cardiac Abnormalities in Wistar Rats: Evidence of Serotonergic, Oxidative, and Inflammatory Pathways. J. Biochem. Mol. Toxicol..

[213] Khalil H.M.A., Mahmoud D.B., El-Shiekh R.A., Bakr A.F., Boseila A.A., Mehanna S., Naggar R.A., Eliwa H.A. (2022). Antidepressant and Cardioprotective Effects of Self-Nanoemulsifying Self-Nanosuspension Loaded with Hypericum Perforatum on Post-Myocardial Infarction Depression in Rats. AAPS PharmSciTech.

[214] Sylvia L.G., Gold A.K., Rakhilin M., Amado S., Modrow M.F., Albury E.A., George N., Peters A.T., Selvaggi C.A., Horick N. (2023). Healthy Hearts Healthy Minds: A Randomized Trial of Online Interventions to Improve Physical Activity. J. Psychosom. Res..

[215] Chen X., Zeng M., Chen C., Zhu D., Chen L., Jiang Z. (2022). Efficacy of Psycho-Cardiology Therapy in Patients with Acute Myocardial Infarction Complicated with Mild Anxiety and Depression. Front. Cardiovasc. Med..

[216] Shi W., Ghisi G.L.M., Zhang L., Hyun K., Pakosh M., Gallagher R. (2022). A Systematic Review, Meta-Analysis, and Meta-Regression of Patient Education for Secondary Prevention in Patients with Coronary Heart Disease: Impact on Psychological Outcomes. Eur. J. Cardiovasc. Nurs..

[217] Zhang W., Zhang H. (2022). Effects of Comprehensive Nursing Intervention Based on Self-Disclosure on Improving Alexithymia in Elder Patients with Coronary Heart Disease. BMC Nurs..

[218] Westas M., Lundgren J., Andersson G., Mourad G., Johansson P. (2022). Effects of Internet-Delivered Cognitive Behavioural Therapy Adapted for Patients with Cardiovascular Disease and Depression: A Long-Term Follow-up of a Randomized Controlled Trial at 6 and 12 Months Posttreatment. Eur. J. Cardiovasc. Nurs..

[219] Chen B., Wen J., You D., Zhang Y. (2024). Implication of Cognitive-Behavioral Stress Management on Anxiety, Depression, and Quality of Life in Acute Myocardial Infarction Patients after Percutaneous Coronary Intervention: A Multicenter, Randomized, Controlled Study. Ir. J. Med. Sci..

[220] Chew T.R., Yeo T.M., Teo J.Y.C., Seah C.W.A., Soh C.S.Q., Meng J., Wang W. (2025). Effectiveness of Psychological Interventions in Reducing Post-Traumatic Stress among Post-Myocardial Infarction Patients: A Systematic Review and Meta-Analysis. Eur. J. Cardiovasc. Nurs..

[221] Carli V., Petros N.G., Hadlaczky G., Vitcheva T., Berchialla P., Bianchi S., Carletto S., Christinaki E., Citi L., Dinis S. (2022). The NEVERMIND E-Health System in the Treatment of Depressive Symptoms among Patients with Severe Somatic Conditions: A Multicentre, Pragmatic Randomised Controlled Trial. EClinicalMedicine.

[222] Ni R., Liu M., Huang S., Yang J. (2022). Effects of eHealth Interventions on Quality of Life and Psychological Outcomes in Cardiac Surgery Patients: Systematic Review and Meta-Analysis. J. Med. Internet Res..

[223] Boszko M., Krzowski B., Peller M., Hoffman P., Żurawska N., Skoczylas K., Osak G., Kołtowski Ł., Grabowski M., Opolski G. (2023). Impact of AfterAMI Mobile App on Quality of Life, Depression, Stress and Anxiety in Patients with Coronary Artery Disease: Open Label, Randomized Trial. Life.

[224] Deng L., Wu Q., Ding F., Liu Y., Shen J., Lin Y., Shi K., Zeng B., Wu L., Tong H. (2022). The Effect of Telemedicine on Secondary Prevention of Atherosclerotic Cardiovascular Disease: A Systematic Review and Meta-Analysis. Front. Cardiovasc. Med..

[225] Kim H., Yoo J., Han K., Jeon H.J. (2022). Physical Activity and Cardiovascular Health in Depression: Links between Changes in Physical Activity and Cardiovascular Risk. Gen. Hosp. Psychiatry.

[226] Terada T., Cotie L.M., Tulloch H., Mistura M., Vidal-Almela S., O’Neill C.D., Reid R.D., Pipe A., Reed J.L. (2022). Sustained Effects of Different Exercise Modalities on Physical and Mental Health in Patients with Coronary Artery Disease: A Randomized Clinical Trial. Can. J. Cardiol..

[227] Reed J.L., Terada T., Cotie L.M., Tulloch H.E., Leenen F.H., Mistura M., Hans H., Wang H.-W., Vidal-Almela S., Reid R.D. (2022). The Effects of High-Intensity Interval Training, Nordic Walking and Moderate-to-Vigorous Intensity Continuous Training on Functional Capacity, Depression and Quality of Life in Patients with Coronary Artery Disease Enrolled in Cardiac Rehabilitation: A Randomized Controlled Trial (CRX Study). Prog. Cardiovasc. Dis..

[228] Cao X., Dong Y., Yu H., Liu X., Gu Y., Song J., Ouyang P., Hong Z. (2024). The Effect of Sitting Baduanjin in Patients with ST-Segment Elevation Acute Myocardial Infarction after Percutaneous Coronary Intervention: A Quasi-Experimental Study. Heart Lung.

[229] Lyu S., Wang H., Wei Q., Cui M., Li Y., Chen Z., Zhang J., Peng F. (2022). Effects of Tai Chi Cardiac Rehabilitation Program on Anxiety and Depression in Patients with Coronary Heart Disease: A Randomized Controlled Clinical Trial. Eur. J. Integr. Med..

[230] Kalra S., Miraj M., Ajmera P., Shaik R.A., Seyam M.K., Shawky G.M., Alasiry S.M., Mohamed E.H., Alasiri H.M., Alzhrani M. (2022). Effects of Yogic Interventions on Patients Diagnosed With Cardiac Diseases. A Systematic Review and Meta-Analysis. Front. Cardiovasc. Med..

[231] Prasad A. (2023). Yogic Exercise—Its Effect on Blood Pressure and Depression Score in Myocardial Infarction. J. Hypertens..

[232] Zhang L., Bao Y., Tao S., Zhao Y., Liu M. (2022). The Association between Cardiovascular Drugs and Depression/Anxiety in Patients with Cardiovascular Disease: A Meta-Analysis. Pharmacol. Res..

[233] Molero Y., Kaddoura S., Kuja-Halkola R., Larsson H., Lichtenstein P., D’Onofrio B.M., Fazel S. (2023). Associations between β-Blockers and Psychiatric and Behavioural Outcomes: A Population-Based Cohort Study of 1.4 Million Individuals in Sweden. PLoS Med..

[234] Leissner P., Mars K., Humphries S., Karlström P., Yndigegn T., Jernberg T., Hofmann R., Held C., Olsson E.M.G. (2024). Short- and Long-Term Effects of Beta-Blockers on Symptoms of Anxiety and Depression in Patients with Myocardial Infarction and Preserved Left Ventricular Function: A Pre-Specified Quality of Life Sub-Study from the REDUCE-AMI Trial. Eur. Heart J. Acute Cardiovasc. Care.

[235] Kim J.H., Song Y.-K., Jang H.Y., Shin J.-Y., Lee H.-Y., Ahn Y.M., Oh J.M., Kim I.-W. (2020). Major Adverse Cardiovascular Events in Antidepressant Users Within Patients With Ischemic Heart Diseases: A Nationwide Cohort Study. J. Clin. Psychopharmacol..

[236] Biffi A., Rea F., Scotti L., Lucenteforte E., Vannacci A., Lombardi N., Chinellato A., Onder G., Vitale C., Cascini S. (2020). Antidepressants and the Risk of Cardiovascular Events in Elderly Affected by Cardiovascular Disease: A Real-Life Investigation From Italy. J. Clin. Psychopharmacol..

[237] Zhang L., Lu N., Liu M. (2023). Selective Serotonin Reuptake Inhibitors Regulate the Interrelation between 5-HT and Inflammation after Myocardial Infarction. BMC Cardiovasc. Disord..

[238] Gutlapalli S.D., Prakash K., Swarnakari K.M., Bai M., Manoharan M.P., Raja R., Jamil A., Csendes D., Desai A., Desai D.M. (2022). The Risk of Fatal Arrhythmias Associated With Sertraline in Patients With Post-Myocardial Infarction Depression. Cureus.

[239] Desai R., Park H., Brown J.D., Mohandas R., Smith S.M. (2022). Norepinephrine Reuptake Inhibitors and Risk of Antihypertensive Treatment Intensification and Major Adverse Cardiovascular Events in Patients with Stable Hypertension and Depression. Pharmacotherapy.

[240] Gutlapalli S.D., Lavu V.K., Mohamed R.A., Huang R., Potla S., Bhalla S., Al Qabandi Y., Nandula S.A., Boddepalli C.S., Hamid P. (2022). The Risk of Fatal Arrhythmias in Post-Myocardial Infarction Depression in Association With Venlafaxine. Cureus.

[241] Kim H., Lee Y.-B., Lee J., Kang D., Kim G., Jin S.-M., Kim J.H., Hur K.Y., Jeon H.J. (2024). Association between Depression, Antidepressant Use, and the Incidence of Atherosclerotic Cardiovascular Diseases. J. Affect. Disord..

[242] Gagné M.-A., Frégeau G., Godbout R., Rousseau G. (2024). The Role of Probiotics in Modulating Myocardial Infarction and Depression-like Symptoms: A Study on Sex-Specific Responses. Biomedicines.

[243] Wang B., Teng Y., Li Y., Lai S., Wu Y., Chen S., Li T., Han X., Zhou H., Wang Y. (2022). Evidence and Characteristics of Traditional Chinese Medicine for Coronary Heart Disease Patients With Anxiety or Depression: A Meta-Analysis and Systematic Review. Front. Pharmacol..

[244] Dehghani F., Vafa M., Ebrahimkhani A., Găman M.-A., Sezavar Seyedi Jandaghi S.H. (2023). Effects of Quercetin Supplementation on Endothelial Dysfunction Biomarkers and Depression in Post-Myocardial Infarction Patients: A Double-Blind, Placebo-Controlled, Randomized Clinical Trial. Clin. Nutr. ESPEN.

